# Zonula occludens-1 at the ischemic blood–brain barrier: mechanisms of disruption, functional significance, and experimental modulation

**DOI:** 10.3389/fcell.2026.1967401

**Published:** 2026-10-21

**Authors:** Bosi Zhou, Zhihan Wang, Xuefeng Jing, Bing Xu, Xue Qin

**Affiliations:** Department of Neurology, Chest Hospital of Shenyang Pharmaceutical University, Liaoning Province, China

**Keywords:** blood–brain barrier, brain ischemia, ischemia–reperfusion, tight junctions, zonula occludens-1 (ZO-1)

## Abstract

Blood–brain barrier (BBB) disruption is a major component of ischemic brain injury, yet the contribution of zonula occludens-1 (ZO-1) is often inferred from changes in total protein abundance. Here we synthesize evidence showing that ZO-1 is instead a dynamically regulated junctional scaffold whose disruption encompasses mechanistically distinct states. Ischemia and reperfusion alter ZO-1 through caveolin-dependent internalization and endolysosomal degradation, phosphorylation and cytoskeletal redistribution, transcriptional and epigenetic regulation, and convergent oxidative, inflammatory, metabolic and multicellular signaling. These mechanisms can impair junctional continuity and paracellular barrier function without necessarily reducing total ZO-1. Conversely, BBB permeability can improve without proportional recovery of ZO-1 abundance, reflecting contributions from transcellular transport, the basal lamina and other neurovascular components. Numerous interventions preserve or restore ZO-1 alongside reduced leakage, edema or hemorrhagic transformation, but most act on upstream injury pathways and do not establish that ZO-1 is necessary for barrier rescue. We therefore propose that ZO-1 should be interpreted as a mechanistically informative readout of endothelial junctional integrity rather than a universal surrogate for BBB recovery or a validated therapeutic target. The available evidence remains predominantly preclinical, while limited clinical studies have evaluated circulating ZO-1 and other tight-junction proteins as biomarkers rather than directly assessing endothelial junctional ZO-1 in the ischemic brain. Translation will therefore require confirmation of ZO-1 molecular states and causal relevance in human BBB systems and patient-level studies, together with clinically relevant reperfusion, comorbidity, and treatment-window paradigms.

## Graphical Abstract

**Figure:**
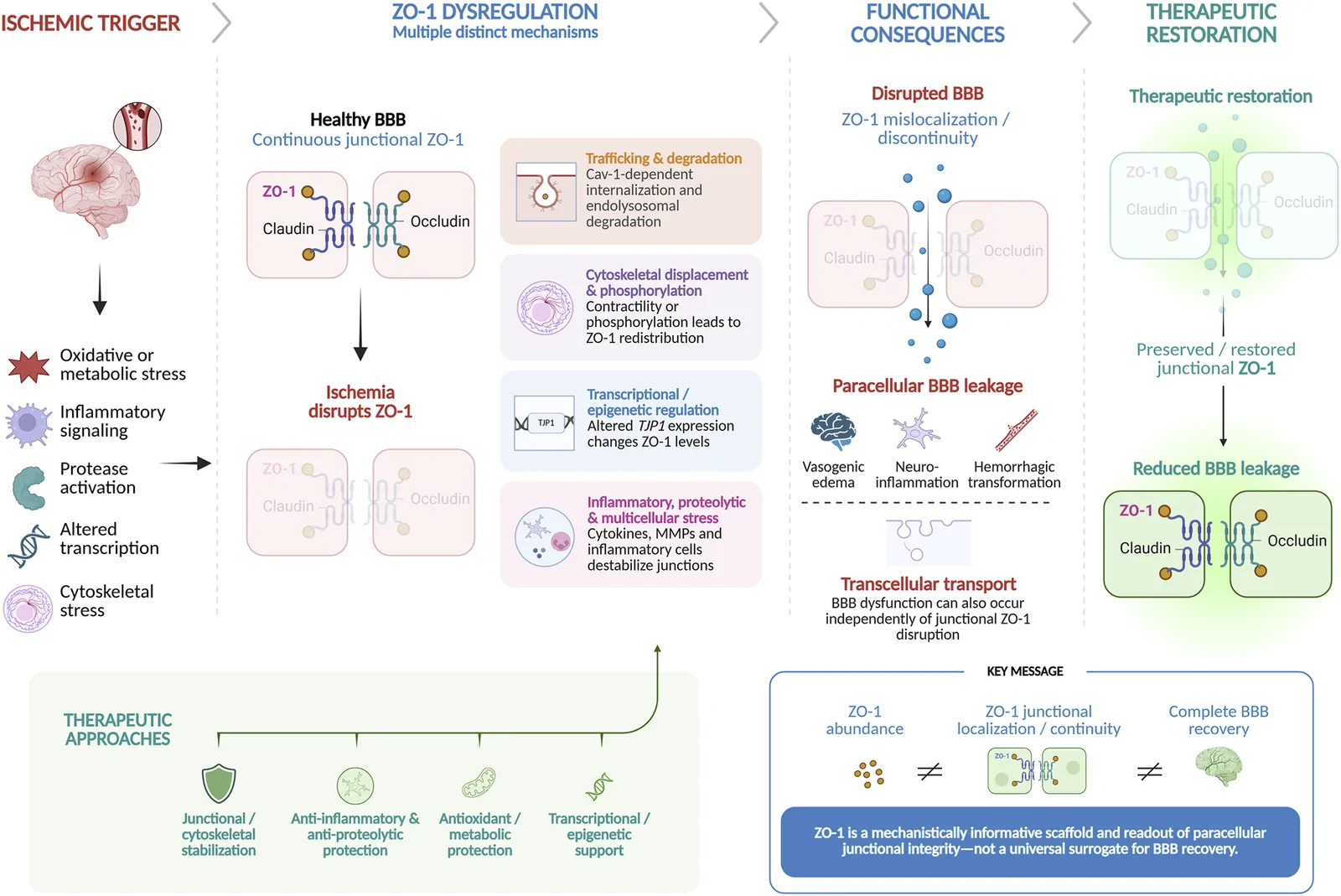

## Highlights

- Ischemia disrupts zonula occludens-1 through distinct molecular mechanisms
- Zonula occludens-1 continuity can be lost without reduced total protein
- Blood–brain barrier recovery can occur without proportional ZO-1 restoration
- Most treatments restore zonula occludens-1 through broader vascular protection
- Zonula occludens-1 is an informative marker, not a complete barrier surrogate

## 1 Introduction

Stroke remains a major cause of death and long-term disability worldwide, with ischemic stroke accounting for the largest proportion of the global stroke burden (GBD 2021 Stroke Risk Factor Collaborators, 2024). Ischemic injury affects not only neurons but also the cerebral microvasculature, leading to progressive dysfunction of the blood brain barrier (BBB) (Jiang et al., 2018). BBB disruption permits circulating molecules and cells to enter the brain and contributes to vasogenic edema, neuroinflammation, hemorrhagic transformation, and poor outcomes (Yang C. et al., 2019).

The BBB is formed primarily by specialized brain endothelial cells whose barrier properties are regulated by their interactions with pericytes, astrocytes, neurons, microglia, the basement membrane, and circulating cells within the neurovascular unit (Jiang et al., 2018). At endothelial cell borders, claudins, occludin, and junctional adhesion molecules interact with cytoplasmic scaffold proteins to restrict paracellular movement between the blood and brain (Stamatovic et al., 2016) (Figure 1). Zonula occludens-1 (ZO-1), encoded by the tight junction protein one gene (TJP1), binds several junctional proteins and connects the tight-junction complex to the actin cytoskeleton (Hartsock and Nelson, 2008). ZO proteins can self-organize through biomolecular phase separation into membrane-associated compartments that concentrate tight-junction components and promote assembly of continuous junctional networks (Beutel et al., 2019). This supramolecular organization provides an additional reason why total ZO-1 abundance alone cannot determine whether the protein is correctly localized and incorporated into a functional junctional structure. Consequently, total ZO-1 abundance alone cannot determine whether the protein is correctly localized and assembled into a continuous junctional structure (Beutel et al., 2019).

**FIGURE 1:**
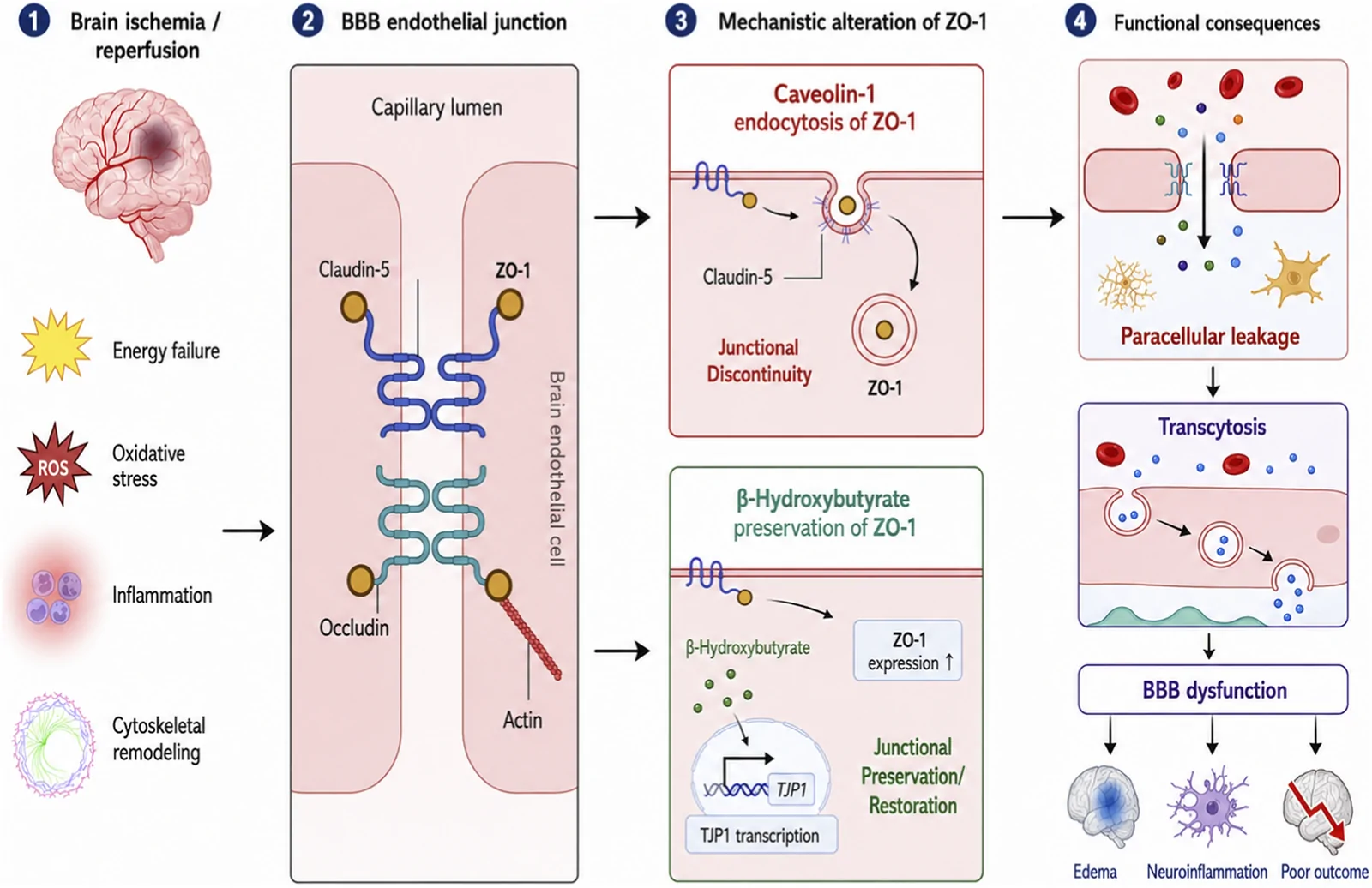
ZO-1 role at the ischemic BBB. Brain ischemia/reperfusion promotes endothelial junctional injury through energy failure, oxidative stress, inflammation, and cytoskeletal remodeling. ZO-1 disruption, including Cav-1–dependent internalization and junctional discontinuity, contributes to paracellular leakage and BBB dysfunction, whereas preservation of TJP1/ZO-1 expression can support junctional restoration.

During brain ischemia and reperfusion, energy failure, oxidative stress, inflammatory signaling, protease activation, and cytoskeletal remodeling contribute to endothelial injury and BBB disruption (Jiang et al., 2018). These processes can influence ZO-1 through distinct molecular mechanisms rather than producing a single uniform pattern of protein loss. For example, caveolin-1 (Cav-1) promoted the endocytosis of ZO-1 and claudin-5 in experimental acute ischemic stroke, directly linking junctional trafficking to increased BBB permeability (Li et al., 2023). In another mechanistic study, β-hydroxybutyrate (BHB) increased endothelial ZO-1 expression by enhancing histone H3 lysine 9 β-hydroxybutyrylation at the TJP1 promoter (Li et al., 2024). BBB permeability can also increase through endothelial transcytosis, which is mechanistically distinct from paracellular leakage caused by tight-junction disruption. Preserved or restored ZO-1 therefore does not, by itself, establish complete recovery of BBB function (Zhou et al., 2021).

Interpretation is further complicated by the use of molecular, histological, imaging, and tracer-based methods that assess different structural or functional aspects of the BBB. ZO-1 findings should therefore be evaluated according to whether a study measured total abundance, endothelial localization, junctional continuity, or an independent functional outcome such as BBB permeability (Sun et al., 2021).

This comprehensive review investigates the molecular and cellular mechanisms that alter ZO-1-containing endothelial junctions during brain ischemia, the relationship between ZO-1 disruption and functional BBB impairment, and the extent to which therapeutic interventions directly restore ZO-1 or normalize it secondarily as part of broader vascular protection. It also considers the methodological and experimental limitations that affect the interpretation and translational relevance of ZO-1 findings.

The literature was identified through targeted searches for peer-reviewed studies addressing ZO-1/TJP1 in cerebral ischemia, ischemia/reperfusion, hypoxia or oxygen–glucose deprivation, BBB dysfunction, junctional regulation, and therapeutic intervention, supplemented by mechanistically relevant studies identified from pertinent reference lists. Studies were prioritized when they directly assessed ZO-1/TJP1 together with endothelial or BBB outcomes. Mechanistic evidence was interpreted qualitatively according to the proximity and specificity of the experimental support. Direct ZO-1 causal evidence required manipulation of TJP1/ZO-1 itself together with assessment of a functional barrier outcome. Pathway-supported ZO-1 regulation referred to studies in which an upstream mechanism was experimentally perturbed, particularly through genetic manipulation, target inhibition, bidirectional manipulation, or rescue/reversal experiments, and the resulting effect on ZO-1 was assessed. Pathway-associated evidence described studies in which modulation of a candidate pathway accompanied ZO-1 and BBB recovery without directly establishing the proposed mechanistic sequence, whereas parallel or associative recovery referred to concurrent improvement of ZO-1 and barrier-related outcomes without specific pathway validation. Interpretation additionally considered whether ZO-1 was measured as total abundance or endothelial junctional localization/continuity, whether independent permeability measurements were included, and whether evidence was obtained *in vitro* or *in vivo*. Importantly, evidence that an upstream pathway regulates ZO-1 was distinguished from evidence that ZO-1 itself is necessary or sufficient for the associated BBB effect. Accordingly, ZO-1 findings are interpreted throughout this review using two complementary dimensions: the molecular state measured; TJP1 transcription, total protein abundance, endothelial localization, junctional continuity, or post-translational/trafficking change, and the strength of evidence linking that state to functional BBB impairment or recovery.

## 2 Molecular dismantling of the ZO-1 junctional complex

Ischemia disrupts ZO-1 through distinct mechanisms, including Cav-1-dependent internalization, endolysosomal or protease-associated loss, cytoskeletal redistribution, phosphorylation-dependent junctional displacement, and altered TJP1 transcription (Li et al., 2023; Zhang et al., 2018; Wu et al., 2018). These mechanisms generate biologically distinct ZO-1 states and should not be collectively described as merely “reduced ZO-1.” In particular, reduced TJP1 transcription or total protein abundance should be distinguished from loss of membrane localization or continuous endothelial-border organization, because the latter can occur despite preserved total ZO-1. Likewise, evidence that an upstream ischemic pathway regulates one of these ZO-1 states does not by itself establish that the ZO-1 alteration mediates the resulting BBB dysfunction. The following sections therefore distinguish molecular abundance, spatial organization, and functional barrier outcomes wherever the available studies permit. Figure 2 summarizes these processes.

**FIGURE 2:**
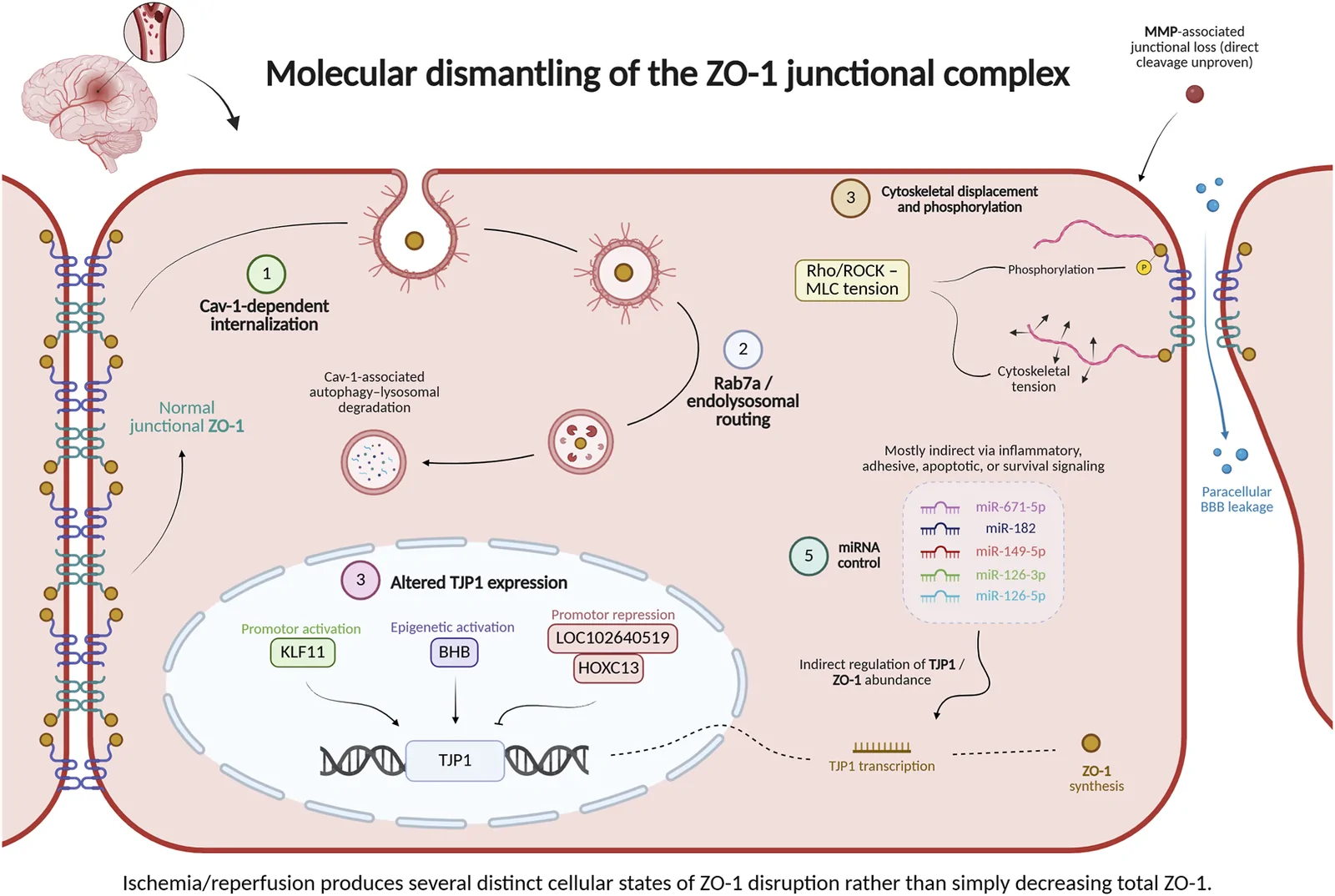
Molecular dismantling of the ZO-1 junctional complex during ischemia/reperfusion. Ischemic injury disrupts junctional ZO-1 through internalization and endolysosomal degradation, cytoskeletal and phosphorylation-dependent displacement, altered TJP1 expression, and microRNA (miRNA)-mediated regulation, collectively contributing to loss of junctional integrity and paracellular BBB leakage.

### 2.1 Caveolin-dependent internalization, autophagy, and endolysosomal degradation

Ischemia can disrupt ZO-1 through endothelial trafficking and degradation pathways rather than through loss of protein abundance alone (Figure 2). In permanent middle cerebral artery occlusion and oxygen–glucose deprivation (OGD), increased Cav-1 was accompanied by reduced membrane-associated ZO-1, increased cytoplasmic redistribution, and greater BBB permeability. Cav-1 knockdown restored ZO-1 and reduced permeability, whereas Cav-1 overexpression produced the opposite effect, supporting Cav-1-dependent ZO-1 internalization (Li et al., 2023).

A reperfusion study extended this mechanism to ZO-1 degradation. Ischemia increased Cav-1 association with ZO-1 and microtubule-associated protein one light chain 3 B and promoted autophagy–lysosomal degradation and abnormal redistribution of ZO-1. Cav-1 knockdown and inhibition of autophagic or lysosomal activity preserved ZO-1, whereas Cav-1 overexpression or autophagy activation weakened its rescue (Li Y. et al., 2025). Endothelial Rab7a deletion similarly preserved ZO-1 abundance and junctional continuity after ischemia and reduced paracellular leakage, supporting additional endolysosomal control of ZO-1-containing junctions (Cottarelli et al., 2025).

However, autophagy was not uniformly associated with ZO-1 degradation. P-glycoprotein silencing, apelin-13 treatment, and pharmacological autophagy induction preserved ZO-1 abundance or junctional localization, whereas autophagy inhibition aggravated ZO-1 loss. Conversely, cyclo-(Phe-Tyr) restored ZO-1 while suppressing excessive autophagy through phosphoinositide 3-kinase (PI3K)/protein kinase B AKT/mammalian target of rapamycin (MTOR) signaling. These studies therefore demonstrate pathway-dependent modulation of ZO-1 but not direct autophagic processing of ZO-1 (Huang et al., 2022; Zhang et al., 2023; Li et al., 2014; Liang et al., 2022).

The strongest evidence supports Cav-1-dependent internalization and Cav-1/Rab7a-associated endolysosomal control of the junctional ZO-1 pool. Autophagy-related effects were context dependent, indicating that autophagic activity can either destabilize or preserve ZO-1-containing junctions depending on the upstream pathway and experimental setting.

### 2.2 Protease, matrix-metalloproteinase, and extracellular-matrix routes to junctional loss

Post-ischemic MMP activation is associated with ZO-1 loss, but the available evidence does not establish direct proteolysis of ZO-1 or distinguish junctional cleavage from broader inflammatory and extracellular-matrix injury (Figure 2). MMP-12 knockdown restored TJP1 transcript, total ZO-1 protein, and capillary immunoreactivity, positioning MMP-12 upstream of junctional-protein loss; however, parallel recovery of messenger RNA (mRNA) argues against interpreting the protein change solely as proteolytic degradation, and no permeability endpoint confirmed functional barrier rescue (Arruri et al., 2022). Conversely, loss of the endogenous inflammatory regulator MCPIP1 increased MMP-3/-9 abundance, further reduced total and vascular-pattern ZO-1, and markedly increased fluorescein isothiocyanate (FITC)-dextran leakage, directly linking pathway-associated ZO-1 loss with BBB dysfunction (Jin et al., 2019a). Yet MMP activity, ZO-1 cleavage, and rescue by MMP inhibition were not tested, while global MCPIP1 deficiency introduces systemic inflammatory confounding. Neither study assessed basement-membrane preservation, so the contribution of extracellular-matrix damage remains unresolved.

Collectively, MMP pathways correlate with ZO-1 preservation and permeability, but direct substrate cleavage and matrix-dependent junctional failure remain unproven.

### 2.3 Cytoskeletal tension, mechano-transduction, phase separation, and control of junctional localization

Ischemia-related contractile, oxidative, and ion-channel signaling weakens the BBB by either disrupting continuous ZO-1 at endothelial borders or reducing its abundance (Figure 2); these changes are not equivalent. Loss of A-kinase anchoring protein 12 (AKAP12) increased Rho activity and myosin light-chain (MLC) phosphorylation, disrupted peripheral ZO-1, and increased endothelial permeability, whereas Rho-associated kinase (ROCK) inhibition improved both junctional organization and barrier function (Seo et al., 2020). A distinct redox mechanism was demonstrated for polymerase delta-interacting protein 2 (POLDIP2); its overexpression increased reactive oxygen species (ROS) and ZO-1 tyrosine phosphorylation, reduced continuous border localization, and impaired the endothelial barrier, while antioxidants reversed phosphorylation and redistribution. Importantly, ZO-1 continuity was lost without a significant reduction in total protein (Wang K. et al., 2025). A potential framework for this abundance–localization dissociation is provided by the emerging mechanobiology of ZO-1. ZO proteins can undergo biomolecular phase separation, generating condensate-like compartments that locally enrich tight-junction proteins and promote their organization into continuous junctional networks (Beutel et al., 2019). Junctional ZO-1 is also mechanically coupled to the actomyosin cytoskeleton: actomyosin-generated forces can regulate ZO-1 conformation and junctional recruitment, while phase-separated ZO-1 clusters can be transported and incorporated into tight junctions through actin-dependent processes (Schwayer et al., 2019; Spadaro et al., 2017). Consistent with a mechano-transductive role in vascular cells, ZO-1 regulates actomyosin organization, intercellular tension, and barrier formation in primary endothelial cells (Tornavaca et al., 2015). Moreover, disruption of β1-integrin–matrix adhesion in primary cerebral microvascular endothelial cells reorganized ZO-1 and other tight-junction proteins away from inter-endothelial borders together with F-actin remodeling, increased MLC phosphorylation, and greater permeability (Izawa et al., 2018). These findings suggest a plausible model in which ischemia-induced changes in cytoskeletal tension, phosphorylation, or endothelial–matrix coupling alter the local assembly and mechanical stabilization of ZO-1 at cell borders, allowing a relatively preserved cellular ZO-1 pool to coexist with loss of junctional continuity. However, ZO-1 phase separation, condensate dynamics, and force-dependent conformational changes have not yet been directly demonstrated in ischemic brain endothelium; their contribution to BBB failure therefore remains a mechanistic hypothesis rather than an established ischemic pathway.

Other contractile studies provide less direct, abundance-centred evidence. Myosin light-chain kinase (MLCK) overexpression reversed Lifibrate-associated recovery of ZO-1 mRNA and permeability, but ZO-1 protein localization was not examined (Duan et al., 2024); Ephrin A1–Eph receptor A4 (EphA4) signaling reduced total cortical ZO-1 through ROCK, but neither junctional localization nor direct permeability was assessed (Chen et al., 2018). Ion-channel evidence was strongest for endothelial leucine-rich repeat-containing protein 8A (LRRC8A). Endothelial deletion reduced leakage, while cellular silencing or blockade restored ZO-1 transcript and fluorescence through with-no-lysine kinase 1 (WNK1)-dependent signaling, although continuity was not quantified (Tian et al., 2025). Global transient receptor potential vanilloid 4 (TRPV4) deletion also reduced leakage but provided only indirect evidence of whole-hemisphere ZO-1 preservation (Tanaka et al., 2020). Collectively, contractile, oxidative, and ion-channel pathways regulate both ZO-1 abundance and its endothelial-border organization. The distinction is important because phosphorylation- or cytoskeleton-associated redistribution can disrupt junctional continuity without reducing total ZO-1.

### 2.4 Transcriptional, epigenetic, and noncoding-RNA control of ZO-1

The strongest evidence for direct regulation of ZO-1 comes from studies of the TJP1 promoter (Figure 2). Endothelial Krüppel-like factor 11 (KLF11) preserved TJP1 mRNA, ZO-1 protein, vascular staining, and BBB function after ischemia. Bidirectional KLF11 manipulation altered TJP1 promoter activity, and deletion of the predicted responsive region abolished this effect, although physical promoter binding and ZO-1 necessity were not tested. BHB similarly restored TJP1 transcription and ZO-1 protein while increasing histone H3 lysine-9 beta-hydroxybutyrylation at the TJP1 promoter. Disruption of ketogenesis or monocarboxylate transporter 1 reduced ZO-1 recovery and worsened leakage, but the histone modification itself was not shown to be necessary (Li et al., 2024; Zhang X. et al., 2020).

A contrasting repressive mechanism involved vascular endothelial growth factor (VEGF), the long noncoding RNA LOC102640519, and homeobox C13 (HOXC13). Silencing LOC102640519 or HOXC13 restored ZO-1 protein and improved transendothelial electrical resistance (TEER) and macromolecular permeability after oxygen–glucose deprivation/reoxygenation (OGD/R). RNA pull-down, chromatin immunoprecipitation, and reporter experiments supported HOXC13-associated repression at the ZO-1 promoter. However, endogenous TJP1 transcription, *in vivo* BBB rescue, and ZO-1-specific mediation were not demonstrated (Wu et al., 2018).

MiRNAs mainly influenced ZO-1 through upstream inflammatory, adhesive, or survival pathways. miR-671–5p restored ZO-1 and endothelial barrier function through a nuclear factor kappa B (NF-κB)-dependent pathway, whereas miR-182 inhibition preserved ZO-1 in association with mammalian target of rapamycin and forkhead box protein O1 (FOXO1) signaling and reduced endothelial apoptosis. MiR-149-5p increased whole-hemisphere TJP1 mRNA and reduced leakage, but ZO-1 protein and the proposed calpain mechanism were not examined. MiR-126-3p and miR-126-5p reduced vascular ZO-1 gaps and immunoglobulin G (IgG) leakage while suppressing vascular cell adhesion molecule-1 or E-selectin, indicating improved junctional continuity without evidence of increased total ZO-1 abundance (Deng et al., 2023; Zhang T. et al., 2020; Forouzandeh et al., 2022; Pan et al., 2020).

Other pathways provided only indirect evidence. Immunoproteasome low-molecular-mass polypeptide 2 silencing restored beta-catenin and ZO-1, but direct beta-catenin regulation of TJP1 was not tested. Likewise, tissue factor pathway inhibitor 2 knockdown preserved total ZO-1 and improved BBB-associated outcomes, although activator protein-1 regulation was demonstrated only at the tissue factor pathway inhibitor 2 promoter, leaving the link to ZO-1 unresolved (Chen et al., 2021; Cao et al., 2024).

Among transcriptional and epigenetic mechanisms, the most resolved evidence supports KLF11-dependent TJP1 promoter activation, BHB-associated promoter modification, and LOC102640519–HOXC13-associated repression. By contrast, most miRNA pathways influence ZO-1 indirectly through inflammatory, adhesive, or survival signaling and affect readouts ranging from TJP1 transcript abundance to junctional continuity.

## 3 Stress pathways that converge on ZO-1

### 3.1 Oxidative and nitrosative stress, mitochondrial failure, and ferroptotic convergence

Oxidative stress altered ZO-1 through junctional disorganization and reduced expression. In endothelial monolayers, hypoxia/reoxygenation increased ROS and nitrotyrosine, reduced ZO-1 membrane enrichment, and caused junctional discontinuities with lower electrical resistance and greater permeability. Diphenyleneiodonium preserved ZO-1 organization and barrier function, implicating a flavoprotein or nicotinamide adenine dinucleotide phosphate oxidase-dependent process, although inhibitor specificity and validation in ischemic brain tissue were limited.

Hypoxia-inducible factor-1α (HIF-1α) and VEGF provided a related expression-level route; hypoxia or VEGF reduced ZO-1 mRNA, protein, and junctional continuity, whereas HIF-1α inhibition or VEGF neutralization partially preserved ZO-1 and reduced leakage (Zehendner et al., 2013; Yeh et al., 2007).

Nitrosative stress was supported by reciprocal *in vivo* manipulation. Post-ischemic S-nitroso glutathione reduced vascular nitrotyrosine, NF-κB activity, MMP-9, BBB leakage, and edema while restoring total ZO-1 protein. Conversely, the peroxynitrite-generating compound 3-morpholinosydnonimine maintained low ZO-1 and worsened leakage. However, direct ZO-1 S-nitrosylation, cleavage, localization, and MMP-dependent degradation were not examined (Khan et al., 2012).

Ferroptosis-associated studies consistently linked antioxidant-pathway recovery to ZO-1 preservation. Sulforaphane restored TJP1 mRNA, total ZO-1, and vascular staining while reducing iron accumulation, ROS, lipid peroxidation, leakage, and edema, although nuclear factor erythroid 2-related factor 2 (NRF2) dependence was not demonstrated (Fan W. et al., 2024). Edaravone dexborneol increased NRF2 nuclear translocation and heme oxygenase-1 (HO-1), cystine/glutamate antiporter, and glutathione peroxidase-4 signaling while preserving ZO-1 and reducing leakage; HO-1 inhibition reversed ZO-1 recovery, but permeability was not reassessed (Xiao et al., 2024). Ginsenoside Rd provided stronger endothelial evidence because the ferroptosis inducer erastin reversed both ZO-1 preservation and functional BBB rescue. Its anti-ferroptotic action depended on neuregulin 1/Erb-B2 receptor tyrosine kinase 4 and PI3K/AKT/MTOR signaling, although ZO-1 was not directly reassessed after pathway inhibition (Hu et al., 2024).

Oxidative and nitrosative stress affect ZO-1 through distinct states: oxidative signaling can redistribute ZO-1 from endothelial borders, whereas hypoxia–VEGF and nitrosative pathways are more often associated with reduced expression or abundance. Ferroptosis-directed studies further support redox control of ZO-1-associated barrier integrity, particularly where pathway inhibition or reversal experiments were performed.

### 3.2 Endothelial apoptosis, inflammasome signaling, and cell-death-associated junctional failure

Ischemia-associated inflammatory signaling was linked to ZO-1 loss through absent in melanoma 2 (AIM2). Global AIM2 deletion after transient cerebral ischemia preserved total and vascular-associated ZO-1 while reducing Evans blue and IgG leakage. In endothelial cells exposed to OGD/R, AIM2 knockdown restored ZO-1 and TEER while reducing signal transducer and activator of transcription 3 (STAT3) phosphorylation, intercellular adhesion molecule 1 expression, and neutrophil adhesion. However, ZO-1 was not assessed after STAT3 inhibition, and neither direct ZO-1 regulation nor its necessity for barrier protection was demonstrated (Xu et al., 2021).

A broader cell-death-associated response was observed after endothelial OGD/R, which reduced ZO-1 alongside NOD-, leucine-rich repeat- and pyrin domain-containing protein 3 (NLRP3) inflammasome activation, ROS production, mitochondrial depolarization, apoptosis, extracellular-matrix remodeling, and MMP-9 elevation. Inhibition of integrin α5 with ATN-161 simultaneously suppressed these changes and partially restored ZO-1 and claudin-5. Because the individual downstream pathways were not manipulated and no permeability or electrical-resistance assay was performed, the study could not determine whether inflammasome activation, mitochondrial injury, or apoptosis caused ZO-1 loss or whether its recovery restored functional BBB integrity (Amruta and Bix, 2021).

These findings place ZO-1 loss within convergent endothelial inflammatory and cell-death responses rather than a single linear pathway. AIM2 suppression was associated with both ZO-1 preservation and functional barrier improvement, whereas integrin α5 inhibition linked ZO-1 recovery to broader suppression of inflammasome activation, mitochondrial dysfunction, and apoptosis.

### 3.3 Endothelial inflammatory signaling as an upstream determinant of ZO-1 stability

Endothelial kinase signaling linked ischemic inflammation to reduced ZO-1 abundance. Nuciferine directly engaged Janus kinase 2 (JAK2), suppressed JAK2/STAT3 activation, and restored ZO-1 and endothelial electrical resistance after OGD. The JAK2 inhibitor AG490 reproduced these effects, supporting pathway involvement, although ZO-1 was not tested as the mediator of barrier rescue.

Similarly, inhibition of sphingosine-1-phosphate receptor 3 (S1PR3) restored TJP1 mRNA, total ZO-1 protein, and vascular-associated staining while reducing Evans blue leakage and edema. Associated changes in p38 mitogen-activated protein kinase, extracellular signal-regulated kinase 1/2, and AKT phosphorylation were not independently manipulated, leaving the downstream mechanism unresolved (Li J. et al., 2025; Fan et al., 2022).

Endothelial-derived soluble suppression of tumorigenicity 2 (sST2) provided genetic evidence for an inflammatory mediator affecting ZO-1-containing barriers. Constitutive sST2 deletion increased stroke-core ZO-1 staining and reduced Evans blue leakage and cerebral swelling without changing lesion volume. However, IgG leakage remained unchanged, indicating that increased ZO-1 staining did not correspond to uniform BBB restoration. Although endothelial cells were the principal source of sST2, the largest transcriptional response to deletion occurred in microglia and macrophages, and neither interleukin-33 (IL-33) sequestration nor direct regulation of endothelial ZO-1 was tested (B et al., 2026).

Inflammatory signaling also converged on protease-associated ZO-1 loss. Histone deacetylase one inhibition increased astrocytic and microglial activation, inflammatory cytokines, ROS, and MMP-2/9 activity while further reducing ZO-1 and increasing Evans blue leakage (Wang et al., 2023). Cyclooxygenase-2 (COX-2) deletion or post-ischemic inhibition similarly linked reduced inflammatory and neutrophil-associated protease activity to preservation of total ZO-1, reduced IgG leakage, and less hemorrhagic transformation (Yang et al., 2020). Delayed antagonism or genetic deletion of the prostaglandin E2 EP1 receptor produced comparable reductions in MMP-3/9 activity and preserved cortical ZO-1. However, none of these studies directly demonstrated MMP-mediated ZO-1 cleavage or isolated endothelial signaling from glial, neuronal, and leukocyte contributions (Frankowski et al., 2015).

A potential stability-related pathway involved endothelial ubiquitin-specific protease 14. Its inhibition after reperfusion preserved cortical and endothelial-associated ZO-1 and reduced Evans blue, IgG, and endothelial dextran leakage. Nevertheless, ZO-1 ubiquitination, binding, protein half-life, and proteasomal flux were not examined; therefore, the findings demonstrate recovery of ZO-1 abundance rather than direct prevention of its degradation. Inflammatory cytokines also decreased after treatment but were not causally connected to endothelial ZO-1 (Hou et al., 2023).

Overall, endothelial inflammatory signaling influences ZO-1 through kinase, lipid-receptor, prostaglandin, proteolytic, and protein-stability pathways. Current evidence is strongest for upstream pathway control of ZO-1 abundance and BBB phenotype, whereas direct regulation of junctional turnover and continuity remains less well resolved.

## 4 Multicellular regulation across the neurovascular unit

### 4.1 Microglial, macrophage, and astrocytic control of endothelial ZO-1

Microglial inflammatory states directly influenced endothelial ZO-1. Poliumoside reduced proinflammatory microglial polarization, interleukin-6, tumor necrosis factor-α (TNF-α), and MMP-9 while preserving vascular ZO-1 and reducing tracer leakage after ischemia. Conditioned medium from activated microglia aggravated endothelial ZO-1 loss and dextran permeability, whereas medium from poliumoside-treated microglia preserved both. JAK/STAT3 signaling regulated microglial activation, but the specific mediator responsible for ZO-1 disruption was not identified (Gao et al., 2025).

Stronger cell-specific evidence came from IL-33–suppression of tumorigenicity 2 (ST2) signaling in monocyte-derived macrophages. ST2 deficiency reduced vascular ZO-1 coverage and increased BBB leakage, whereas IL-33 protection required microglia and macrophages. Bone-marrow chimera, adoptive-transfer, and conditioned-medium experiments showed that ST2-competent macrophages preserved endothelial ZO-1 and reduced permeability. However, the protective macrophage-derived factor remained unidentified (Wang et al., 2024).

Astrocytes mediated both damaging and protective effects. Activated microglia promoted a complement component 3 days-positive inflammatory astrocyte phenotype through interleukin-1α, TNF-α, and complement component C1q. Conditioned medium from these astrocytes reduced endothelial ZO-1, while semaglutide limited microglial activation, inflammatory astrocyte formation, vascular ZO-1 gaps, and BBB leakage. Conversely, Sonic hedgehog increased angiopoietin-1 (Ang-1) production, particularly in astrocytes, and restored endothelial TJP1 mRNA and ZO-1 protein. Ang-1neutralization blocked this transcriptional response, although direct astrocyte-to-endothelial transfer was not tested (Zhang et al., 2022; peng et al., 2013).

These studies demonstrate that endothelial ZO-1 is regulated by bidirectional signaling from microglia, macrophages, and astrocytes. The most informative experiments used cell-specific or conditioned-medium approaches to distinguish neurovascular-unit signaling from endothelial-intrinsic responses, although the final soluble mediators were not always identified.

### 4.2 Leukocyte-endothelial and systemic inflammatory interactions

Peripheral leukocytes influenced endothelial ZO-1 through inflammatory signaling. Increasing miR-29 b in leukocytes reduced C1q and tumor necrosis factor-related protein 6, interleukin-1β (IL-1β), and endothelial apoptosis while restoring total endothelial ZO-1 in a leukocyte–endothelial coculture. The intervention also increased brain ZO-1 and reduced edema after ischemia. However, direct miR target binding was not validated, and functional BBB permeability was not measured (Ma et al., 2022).

Leptin linked endothelial stabilization to reduced neutrophil entry. Administered at reperfusion, leptin restored endothelial ZO-1, reduced MMP-9 activity and Evans blue leakage, and limited neutrophil adhesion and cerebral infiltration. Because circulating neutrophil numbers and motility were not reduced, decreased brain entry was attributed mainly to improved barrier restriction. Nevertheless, leptin-receptor signaling, MMP-9 dependence, and ZO-1 necessity were not tested (Hung et al., 2020). Endothelial chemokine regulation also altered macrophage recruitment and ZO-1 continuity. Endothelial-specific atypical chemokine receptor one overexpression preserved continuous vessel-associated ZO-1, reduced leakage of small and large tracers, and limited macrophage accumulation after ischemia. The proposed mechanism involved sequestration of excess chemokines, but the relevant chemokine ligands were not identified, and direct regulation of ZO-1 was not demonstrated (Guo et al., 2026).

Similarly, blockade of C-X-C chemokine receptor type 4 reduced myeloperoxidase-positive inflammatory-cell accumulation, selected cytokines, ZO-1 junctional gaps, BBB leakage, and edema after permanent ischemia. This study specifically demonstrated improved ZO-1 continuity rather than increased total abundance. However, it did not determine whether protection resulted from direct endothelial receptor inhibition or indirectly from reduced leukocyte recruitment and cytokine exposure (Huang et al., 2013).

These studies place leukocyte–endothelial communication upstream of both ZO-1 abundance and junctional continuity. Leukocytic miR-29b altered endothelial ZO-1 through an inflammatory coculture pathway, leptin stabilized the barrier while restricting neutrophil entry, and chemokine-pathway interventions preserved ZO-1 while reducing macrophage or inflammatory-cell recruitment. These findings link immune trafficking to ZO-1-containing junctions, but none establishes the specific leukocyte-derived mediator, direct molecular regulation of ZO-1, or ZO-1 necessity for BBB protection.

### 4.3 Stem-cell, extracellular-vesicle, and trophic communication

Stem-cell interventions preserved ZO-1 mainly through broad paracrine or systemic effects. Human umbilical cord-derived mesenchymal stem cells (MSCs), administered 24 h after reperfusion, increased total brain ZO-1 and reduced Evans blue leakage. Silencing tumor necrosis factor receptor 1 (TNFR1) in the transplanted cells weakened both effects, linking an MSC-associated factor to BBB protection, although TNFR1 secretion, endothelial target engagement, and ZO-1-specific regulation were not demonstrated. A membrane-free adipose-stem-cell extract similarly preserved cortical ZO-1 and reduced leakage and edema while suppressing Toll-like receptor 4 (TLR4), NF-κB, inflammasome, and MMP-9 signaling. However, these pathways changed in parallel, and the effective regimen was prophylactic rather than post-ischemic (Liu et al., 2024; Ryu et al., 2022).

Extracellular vesicles (EVs) provided more specific evidence of intercellular communication. EVs from interleukin-4-stimulated microglia delivered miR-23a-5p to astrocytes, directly targeted tumor necrosis factor mRNA, and altered astrocyte-to-endothelial signaling. EV treatment reduced vascular ZO-1 gaps and IgG leakage, whereas miR-23a-5p knockdown weakened astrocytic molecular changes, endothelial ZO-1 rescue, and *in vivo* BBB protection. The downstream TNF–MMP-3–NF-κB pathway was not independently validated, and ZO-1 necessity remained untested (Pan et al., 2024).

Vesicle activity also depended on donor-cell state. Microvesicles from unstressed endothelial cells promoted astrocyte survival and subsequently increased endothelial ZO-1 and reduced dextran permeability. In contrast, microvesicles released during OGD impaired astrocytes, reduced and fragmented endothelial ZO-1, and increased permeability. Corresponding beneficial or harmful effects on Evans blue leakage occurred *in vivo*, although vascular ZO-1 was not measured and neither the vesicular cargo nor the astrocyte-derived endothelial mediator was identified. Plant-derived exosome-like nanoparticles also restored total ZO-1 and reduced leakage while delivering miR-5266 capable of binding the MMP-9 mRNA untranslated region; however, cargo necessity and endothelial uptake were not established (Pan et al., 2016; Cai et al., 2022).

Trophic communication was less directly resolved. Combined salvianolic acids and Panax notoginseng saponins restored total ZO-1 and reduced BBB leakage alongside preservation of endothelial cells, pericytes, astrocytic end-feet, and brain-derived neurotrophic factor-associated signaling. Because no trophic pathway, glial population, or donor-cell factor was manipulated, ZO-1 recovery could not be separated from broader neurovascular-unit protection (Chen et al., 2024).

Stem-cell and extracellular-vesicle studies support indirect regulation of endothelial ZO-1 through paracrine signaling, transferred miRNAs, and astrocyte-mediated communication. Mechanistic resolution was greatest for microglial extracellular-vesicle miR-23a-5p, whereas most other interventions produced broader neurovascular protection with parallel ZO-1 recovery.

## 5 Context-dependent uncoupling of ZO-1 and BBB function

### 5.1 Reperfusion, recanalization, recombinant tissue plasminogen activator, and hemorrhagic transformation

Reperfusion did not produce a uniform ZO-1 response. After transient middle cerebral artery occlusion, microvascular ZO-1 mRNA, protein, and continuity showed early and delayed reductions that coincided with BBB-leakage peaks at approximately 3 and 72 h. A comparison of reperfused and permanent ischemia likewise found lower and more fragmented vascular ZO-1 after reperfusion, although different occlusion sites and surgical models prevented recanalization from being isolated as the cause. Conversely, recanalization after 3 days of ischemia restored ZO-1 and reduced leakage in surviving penumbra while increasing leakage in the ischemic core. Fibroblast growth factor receptor one inhibition weakened penumbral ZO-1 recovery, implicating PI3K/AKT signaling without demonstrating direct ZO-1 regulation (Jiao et al., 2011; Wang RF. et al., 2025; Zheng et al., 2023).

Recombinant tissue plasminogen activator (rt-PA) could amplify endothelial injury independently of thrombolysis. In primary endothelial monolayers, rt-PA did not disrupt ZO-1 under normoxia but increased endocytic activity and albumin transport. During hypoxia/reoxygenation, it aggravated ZO-1 discontinuity, nearly halved the residual electrical resistance, and further increased small-solute and albumin permeability, indicating combined paracellular and transport-associated dysfunction (Hiu et al., 2008). In a separate ischemia-like model, inhibition of NLRP3 preserved ZO-1 signal, endothelial survival, and monolayer impedance during rt-PA exposure. However, complete inflammasome activation, tracer permeability, reperfusion, and hemorrhage were not assessed (Bellut et al., 2022).

The strongest evidence that ZO-1 contributes to therapeutic barrier reinforcement came from all-trans retinoic acid. Retinoic acid receptor-α inhibition prevented induction of ZO-1 and vascular endothelial cadherin and abolished endothelial permeability protection, while ZO-1 knockdown partially reversed the benefit. *In vivo*, post-ischemic retinoic acid preserved ZO-1 during rt-PA exposure and reduced Evans blue leakage and intracerebral hemorrhage. Nevertheless, ZO-1 necessity was demonstrated only in cultured endothelial cells, not in the hemorrhagic-transformation model (Kong et al., 2015). Poly (adenosine diphosphate-ribose) polymerase inhibition with PJ34 also preserved ZO-1 and reduced rt-PA-associated hemorrhage without changing infarct volume or edema, supporting a vascular effect, although pathway activation and direct ZO-1 stabilization were not measured (Teng et al., 2013).

Other adjunctive studies provided pathway-level rather than ZO-1-specific evidence. Glycogen synthase kinase-3β (GSK-3β) inhibition increased β-catenin and total ZO-1 while reducing rt-PA-associated leakage and hemorrhage, but β-catenin dependence, endothelial localization, and ZO-1 necessity were not tested (Wang et al., 2016). In a true thromboembolic model, early 2-(2-benzofuranyl)-2-imidazoline treatment allowed delayed rt-PA recanalization with greater ZO-1 preservation and less leakage, accompanied by lower MMP-2/9 activity. However, hemorrhagic transformation was not measured, and neither MMP-mediated ZO-1 degradation nor cargo-specific regulation was demonstrated (Zhang L. et al., 2020).

Therefore, reperfusion and recanalization can either aggravate or restore ZO-1-containing junctions depending on timing, tissue region, and pre-existing ischemic injury. Rt-PA adds direct endothelial toxicity, worsening ZO-1-associated paracellular failure under ischemic conditions while also affecting transport pathways independently of visible ZO-1 disruption. Retinoic acid provides the strongest evidence that ZO-1 functionally contributes to barrier rescue, whereas other interventions preserve ZO-1 in parallel with reduced leakage or hemorrhage. Most rt-PA studies used mechanical or permanent occlusion rather than thrombolysis-mediated recanalization, and none established ZO-1 necessity for preventing hemorrhagic transformation *in vivo*.

### 5.2 Paracellular leakage, transcellular transport, edema, and basal-lamina failure

Paracellular leakage can arise from loss of junctional ZO-1 organization without reduced total protein. In primary brain endothelial monolayers, hypoxia redistributed ZO-1 from continuous cell borders into a diffuse, discontinuous pattern while increasing sucrose permeability 2.6-fold, despite preserved total ZO-1 and cell viability. Reoxygenation restored junctional localization and partially reduced permeability. These findings identify ZO-1 continuity, rather than abundance alone, as the more informative structural correlate of paracellular failure, although actin remodeling and ZO-1 causality were not directly tested (Mark and Davis, 2002). This dissociation between total abundance and junctional continuity is compatible with the phase-separation and mechano-transduction framework discussed in Section 2.3, in which changes in actomyosin mechanics or endothelial–matrix coupling could redistribute an existing ZO-1 pool without requiring net protein loss. Direct measurement of ZO-1 condensate behavior or mechanical state during ischemic BBB disruption will be required to test this explanation.

Edema reflected combined junctional, proteolytic, and water-balance disturbances. Postischemic albumin increased MMP-9, further reduced total ZO-1, occludin, and claudin-5, and aggravated Evans blue leakage and cerebral edema despite increasing plasma colloid osmotic pressure. MMP inhibition partially restored junctional proteins, but functional leakage and edema were not reassessed, and direct ZO-1 cleavage was not demonstrated (Li et al., 2022). Melatonin similarly preserved ZO-1 while reducing MMP-9, astrocytic aquaporin-4 (AQP4), perivascular swelling, Evans blue leakage, and edema. However, the parallel improvement of several neurovascular components prevented attribution of edema reduction specifically to ZO-1 preservation (Lee et al., 2024).

Basal-lamina and junctional preservation were partly dissociable from total ZO-1 recovery. Chlorpromazine plus promethazine reduced Evans blue leakage and brain edema under both hypothermic and normothermic conditions while preserving laminin and several junctional proteins and reducing MMP-2/9 and AQP4/9. Significant ZO-1 recovery, however, occurred mainly when drug-induced hypothermia was permitted; normothermic treatment improved BBB function without clearly restoring ZO-1. This discordance indicates that reduced permeability and edema can result from protection of other junctional, basal-lamina, proteolytic, or water-transport components even when total ZO-1 remains low. Neither basal-lamina degradation nor the relative paracellular and transcellular contributions was directly resolved (Li F. et al., 2019).

So, BBB leakage and edema cannot be interpreted solely from total ZO-1 abundance. Paracellular dysfunction was most clearly associated with displacement and discontinuity of junctional ZO-1, whereas edema additionally involved MMPs, astrocytic aquaporins, perivascular swelling, and basal-lamina integrity. Functional barrier improvement could occur without clear ZO-1 restoration, demonstrating that ZO-1 is one component of a broader neurovascular permeability system. None of the studies directly separated paracellular from transcellular transport *in vivo* or established that ZO-1 preservation was necessary or sufficient to prevent edema or basal-lamina failure.

### 5.3 Angiogenesis and vascular repair: Restorative junctions or permissive leak?

Angiogenesis and ZO-1 restoration were often reported together, but their causal relationship remained uncertain. Nicotinamide adenine dinucleotide phosphate administered at reperfusion preserved ZO-1, improved tight-junction ultrastructure, and reduced Evans blue leakage and edema at 24 h while suppressing inflammasome-, MMP-9-, and Cav-1-associated responses. Treatment later increased HIF-1α, VEGFA, and vascular density at day 7, but ZO-1 and permeability were not reassessed at this reparative stage. The study therefore demonstrates temporally separated acute junctional protection and later angiogenesis rather than maturation of newly formed ZO-1-containing vessels (Xu et al., 2026).

Cystathionine β-synthase (CBS)-generated hydrogen sulfide produced concurrent vascular and junctional recovery. Repeated L-cysteine treatment restored ZO-1 and claudin-5, reduced Evans blue leakage and edema, and increased vascular endothelial growth factor receptor 2 (VEGFR2), microvascular density, cerebral blood flux, and angiogenesis. CBS inhibition abolished ZO-1 and BBB rescue, supporting hydrogen-sulfide-pathway involvement. However, VEGFR2 inhibition was evaluated for angiogenesis and broader recovery but not for ZO-1 or permeability. Thus, angiogenesis and junctional restoration remained parallel branches of hydrogen sulfide signaling rather than a demonstrated VEGFR2–ZO-1 sequence (Liang et al., 2026).

Other growth-factor pathways supported vascular stabilization without proving that neovascularization was required. Hypoxia-inducible factor prolyl-hydroxylase inhibition induced VEGF and erythropoietin (EPO)-associated signaling while preserving membrane-localized ZO-1, reducing junctional gaps, and limiting vasogenic edema. Recombinant Ang-1 similarly restored microvascular ZO-1 mRNA and protein and reduced leakage from 12 h to 7 days while suppressing protein kinase C-α and MLC phosphorylation. Ang-1 did not improve regional cerebral blood flow, suggesting direct junctional stabilization rather than permeability improvement secondary to reperfusion or vessel growth. However, neither study established which trophic mediator controlled ZO-1 or whether preserved ZO-1 was required for the barrier benefit (Reischl et al., 2014; Yu et al., 2012).

Overall, the available evidence favors a model in which successful vascular repair requires angiogenesis to be coupled to junctional stabilization rather than assuming that increased vessel density indicates BBB restoration. Hydrogen sulfide and nicotinamide adenine dinucleotide phosphate promoted both angiogenic and ZO-1-associated outcomes, but neither study demonstrated that newly formed vessels acquired mature, continuous ZO-1 junctions or that angiogenesis caused reduced leakage. Growth-factor interventions could preserve ZO-1 and limit edema independently of measurable perfusion improvement, supporting a distinct endothelial-maturation component. No study directly compared mature non-leaky neovessels with immature permissive vessels or established ZO-1 as necessary for functional postischemic vascular repair.

### 5.4 Age, sex, comorbidities, exposures, model choice, and treatment timing

Evidence for age and sex as independent modifiers of ZO-1 was limited because most studies used young male animals. In 24-month-old ovariectomized female rats, ischemia reduced ZO-1 mRNA and protein and increased BBB leakage and edema. GSK-3 inhibition restored ZO-1 protein without restoring its mRNA, suggesting post-transcriptional preservation, while estrogen receptor-β activation produced similar protein recovery. However, age, sex, and estrogen loss could not be separated because young females, aged males, and non-ovariectomized controls were absent, and all treatments were administered prophylactically (Xiao et al., 2017).

Metabolic and systemic comorbidities generally increased junctional vulnerability. Chronic type 1 diabetes accelerated ZO-1 loss and hemorrhagic transformation after prolonged ischemia and reperfusion, whereas acute hyperglycemia rapidly displaced ZO-1 from the endothelial membrane during the first 30 min of reperfusion. Acute iron overload further aggravated ZO-1 loss, Evans blue leakage, and severe hemorrhage under hyperglycemia, while deferoxamine administered at reperfusion reversed these outcomes. Hyperlipidemia also reduced ZO-1 expression and increased leakage, but the protective intervention was given mainly before ischemia. In chronic renal failure, cilostazol increased ZO-1 and reduced leakage; however, the absence of isolated renal-failure and stroke groups prevented a specific comorbidity interaction from being demonstrated (Zhang et al., 2018; Lai et al., 2022; Wu et al., 2022; Shi et al., 2026; Sun et al., 2024).

Environmental exposure provided additional evidence of altered susceptibility. Bisphenol A dose-dependently aggravated ZO-1 and claudin-5 loss, endothelial apoptosis, reduced electrical resistance, and increased dextran permeability during OGD/R. Inhibition of C-X3-C motif chemokine receptor one partially rescued the junctional and functional changes, supporting pathway involvement. Nevertheless, the evidence was confined to endothelial and endothelial–astrocyte culture systems, without an *in vivo* exposure model or demonstration that ZO-1 mediated the permeability effect (Zhou et al., 2026).

Model choice and treatment timing also influence interpretation. Chronic disease models cannot be equated with acute metabolic perturbations, and disease-specific comparison groups are required to distinguish comorbidity effects from greater overall ischemic injury. In addition, several interventions were administered prophylactically, intracerebroventricularly, or at the onset of reperfusion; such designs support preconditioning or very early vascular rescue more strongly than treatment of an established clinical stroke.

Collectively, age, hormonal status, diabetes, hyperglycemia, hyperlipidemia, renal failure, iron loading, and environmental exposure can alter the magnitude or timing of ZO-1 disruption. The strongest evidence concerns metabolic stress, which accelerated ZO-1 loss or redistribution and amplified leakage and hemorrhage. However, age and sex effects remain poorly isolated, environmental evidence is largely *in vitro*, and several comorbidity models lack appropriate comparison groups. The predominance of young male animals, acute disease simulations, tissue-level ZO-1 measurements, and prophylactic treatment schedules limits translation, and no study established that these modifying effects on BBB outcomes were mediated specifically by ZO-1.

### 5.5 A state-based framework for interpreting ZO-1 and BBB dysfunction

The evidence reviewed above supports a state-based interpretation of ZO-1 rather than a linear model in which reduced ZO-1 expression is assumed to indicate proportionally greater BBB disruption. TJP1 transcription, total ZO-1 protein abundance, endothelial membrane localization, junctional continuity, and post-translational or trafficking changes describe different molecular states and provide different levels of information about barrier integrity. Their interpretation should therefore depend both on the ZO-1 endpoint measured and on whether an independent functional BBB outcome was assessed.

Reduced TJP1 transcription or total ZO-1 abundance indicates contraction of the available cellular ZO-1 pool but does not, by itself, demonstrate loss of functional endothelial junctions. Tissue homogenates are particularly limited because they cannot establish whether the measured change occurred within brain endothelium or whether the remaining protein retained continuous junctional organization. Transcriptional regulation by KLF11, β-hydroxybutyrate-associated histone modification, and LOC102640519–HOXC13 signaling provides comparatively strong evidence that ischemia-related pathways can alter TJP1 expression (Li et al., 2024; Wu et al., 2018; Zhang X. et al., 2020), but these studies do not establish that the resulting change in ZO-1 abundance is necessary or sufficient for the associated BBB phenotype.

Altered localization represents a distinct state. ZO-1 may be internalized, redistributed into the cytoplasm, or displaced from endothelial borders through trafficking, phosphorylation, or cytoskeletal mechanisms without a proportional decrease in total protein. Cav-1 manipulation provides pathway-supported evidence for ischemia-associated internalization of ZO-1, while oxidative and contractile signaling can disrupt peripheral ZO-1 organization through phosphorylation and cytoskeletal remodeling (Li et al., 2023; Seo et al., 2020; Wang K. et al., 2025). Such findings are mechanistically more informative than total abundance measurements because they directly address the junctional pool of ZO-1. Nevertheless, even bidirectional manipulation of an upstream regulator demonstrates control of ZO-1 organization rather than proving that ZO-1 itself mediates the accompanying permeability change.

Loss of junctional continuity or development of endothelial-border gaps represents the ZO-1 state most consistently associated with paracellular barrier dysfunction. In hypoxic brain endothelial monolayers, ZO-1 changed from a continuous border pattern to diffuse and discontinuous localization while sucrose permeability increased despite preservation of total protein; reoxygenation restored junctional localization and partially improved permeability (Mark and Davis, 2002). Similarly, several ischemic models associate fragmented or discontinuous vascular ZO-1 with increased tracer leakage. These findings support junctional continuity as a more functionally informative structural correlate than total abundance alone, but most remain correlative because ZO-1 itself was not manipulated.

The reverse dissociation is equally important. Restoration of ZO-1 abundance does not necessarily establish complete BBB recovery, and functional barrier improvement can occur without proportional ZO-1 restoration. This reflects the contribution of other determinants of BBB function, including claudin-5 and occludin organization, endothelial transcytosis, basal-lamina integrity, endothelial survival, proteolytic activity, astrocytic water handling, and other components of the neurovascular unit. Thus, preserved or restored ZO-1 should be interpreted most strongly as evidence of junctional preservation when endothelial localization or continuity is demonstrated together with an independent permeability endpoint, rather than as a universal surrogate for complete BBB restoration.

The causal evidence should also be distinguished explicitly from these structural associations. ZO-1-specific causal evidence requires direct manipulation of TJP1/ZO-1 together with measurement of barrier function. Such evidence remains uncommon in the ischemic BBB literature. ZO-1 knockdown partially reversed retinoic-acid-mediated endothelial barrier protection, providing evidence that ZO-1 contributed functionally to the response; however, this dependence was demonstrated in cultured endothelial cells and was not established in the corresponding *in vivo* hemorrhagic-transformation model (Kong et al., 2015). A larger body of literature instead provides pathway-supported evidence, in which manipulation of an upstream mechanism alters ZO-1 together with BBB function without testing whether ZO-1 mediates that functional effect. Studies in which treatment simply restores ZO-1 while leakage, edema, or other BBB-associated outcomes improve in parallel should be regarded as associative evidence. Table 1 summarizes this framework and the interpretation appropriate to each ZO-1 state.

**TABLE 1:** Conceptual framework for interpreting ZO-1 states at the ischemic blood–brain barrier.

ZO-1 state	What the measurement establishes	Functional interpretation	Main limitation	Appropriate evidence language
Reduced TJP1 mRNA	Altered transcription or transcript stability	May reduce the protein pool available for junction assembly	Does not establish protein loss, endothelial localization, junctional disruption, or permeability change	“Transcriptional regulation of TJP1”
Reduced total ZO-1 protein	Lower overall protein abundance in the sampled cells/tissue	May be compatible with weakened junctional capacity	Does not determine endothelial specificity, membrane localization, continuity, or functional BBB integrity	“Reduced/restored ZO-1 abundance”
Reduced membrane-associated ZO-1 or cytoplasmic redistribution	Loss of junctional enrichment/altered trafficking	More directly consistent with junctional destabilization than total abundance alone	May coexist with preserved total protein; functional significance requires independent barrier testing	“ZO-1 redistribution” or “loss of junctional localization”
Fragmented/discontinuous endothelial-border ZO-1	Loss of continuous spatial organization at endothelial contacts	Most directly associated with impaired paracellular barrier organization	Association does not establish ZO-1 necessity unless ZO-1 itself is manipulated	“Loss/restoration of junctional continuity”
Altered ZO-1 phosphorylation/trafficking/degradation	A specific regulatory state or process when directly demonstrated	May explain altered localization or abundance	Upstream pathway manipulation alone does not prove that ZO-1 mediates BBB dysfunction	“Pathway-supported ZO-1 regulation”
ZO-1 improvement + BBB improvement without mechanistic linkage	Concurrent molecular and functional recovery	Compatible with junctional rescue	Cannot distinguish ZO-1 mediation from broader endothelial/neurovascular protection	“Associative” or “parallel recovery”
Direct TJP1/ZO-1 manipulation + barrier outcome	Functional contribution of ZO-1 itself	Supports causal involvement under the tested conditions	Causality remains model-, cell-, time-, and context-specific	“ZO-1-specific causal evidence”

### 5.6 Model- and method-dependent interpretation of ZO-1 at the ischemic BBB

Interpretation of ZO-1 depends not only on the molecular state measured but also on the experimental model and detection approach used (Table 2). No single model reproduces all components of the ischemic BBB, and no single ZO-1 assay establishes both junctional structure and functional barrier integrity. Accordingly, apparent differences among studies may reflect differences in cellular context, ischemia/reperfusion paradigm, sampling region or time point, and the specific ZO-1 endpoint assessed rather than true biological disagreement.

**TABLE 2:** Strengths and limitations of experimental models and detection approaches for interpreting ZO-1-related BBB dysfunction.

Model or method	What it can establish	Principal strength	Principal limitation for ZO-1 interpretation
*In vivo* focal/transient/permanent or global ischemia models	ZO-1 changes within integrated ischemic/reperfusion injury	Preserves neurovascular, inflammatory, systemic, and regional context	Whole-tissue outcomes cannot by themselves identify endothelial ZO-1 localization or causality
Whole-brain/cortical/peri-infarct tissue qPCR or Western blot	TJP1 transcript or total ZO-1 abundance	Quantitative molecular comparison	No direct information on endothelial specificity, membrane localization, or junctional continuity
Isolated cerebral microvessels	Vascular-enriched transcript/protein changes	Greater vascular specificity than whole tissue	Abundance measurements still do not establish junctional localization or cellular mechanism
Vascular immunofluorescence/immunohistochemistry	Endothelial-associated distribution, coverage, gaps, continuity	Provides spatial information unavailable from homogenates	Structural association; fluorescence recovery does not demonstrate ZO-1-mediated barrier rescue
Endothelial OGD, hypoxia, or OGD/R monolayers	Cell-autonomous ZO-1 expression, redistribution, signaling, and pathway manipulation	High mechanistic control; can combine localization with TEER/permeability	Excludes circulating and multicellular neurovascular interactions and cannot by itself establish an *in vivo* mechanism
Endothelial–astrocyte/pericyte coculture or multicellular BBB systems	Intercellular regulation of endothelial ZO-1 and barrier properties	Incorporates selected neurovascular interactions and functional assays	Still lacks the full systemic and regional context of cerebral ischemia/reperfusion
TEER/impedance	Functional change in endothelial barrier resistance	Repeated quantitative functional measurement	Not ZO-1-specific and does not identify the molecular route of barrier failure
Tracer permeability (dextran, sucrose, albumin, IgG, Evans blue, *etc.*)	Functional barrier leakage	Directly demonstrates altered permeability	Does not automatically distinguish paracellular from transcellular transport or prove ZO-1 mediation
Junctional ZO-1 continuity/gap analysis	Spatial integrity of endothelial-border ZO-1	More functionally informative than total abundance for paracellular junction organization	Remains associative unless ZO-1 itself is manipulated
Electron microscopy/ultrastructure	Tight-junction morphology, endothelial/basal-lamina injury	High structural resolution	Does not identify ZO-1-specific molecular regulation or causality
Genetic/pharmacological pathway manipulation	Whether an upstream pathway contributes to ZO-1/BBB changes	Strengthens pathway-level causal inference	Does not establish ZO-1 as the mediator unless TJP1/ZO-1 itself is tested
Direct TJP1/ZO-1 loss- or gain-of-function with barrier assay	Functional contribution of ZO-1 under the tested conditions	Strongest evidence for ZO-1-specific causality	Context-specific and currently uncommon in ischemic BBB studies

*In vivo* cerebral ischemia models provide the most integrated setting for evaluating ZO-1 in the context of reperfusion, circulating cells, glial responses, basal-lamina injury, edema, and other components of the neurovascular unit. Their interpretive limitation is that whole-brain, hemispheric, cortical, or peri-infarct measurements can obscure endothelial specificity and spatial organization. Western blotting or transcript analysis of tissue therefore establishes changes in total ZO-1 protein or TJP1 mRNA within the sampled tissue but cannot determine whether ZO-1 remains localized continuously at endothelial borders. Isolated cerebral microvessels increase vascular specificity compared with whole-tissue homogenates, but abundance measurements from these preparations still do not by themselves resolve junctional continuity or identify the cellular mechanism responsible for the change.

Immunofluorescence or immunohistochemical analysis provides complementary spatial information. Colocalization of ZO-1 with endothelial markers and assessment of continuous *versus* fragmented vascular staining can distinguish junctional organization from total abundance and is therefore particularly informative when ischemia alters localization without reducing total protein. However, fluorescence intensity, vascular coverage, or gap formation remains a structural readout; spatial association with leakage does not demonstrate that the observed ZO-1 alteration caused the permeability change. Its functional interpretation is strengthened when imaging is combined in the same experiment with an independent tracer-permeability endpoint and, ideally, experimental manipulation of the proposed pathway or ZO-1 itself.

Endothelial monoculture models exposed to hypoxia, oxygen–glucose deprivation, or reoxygenation provide greater experimental control and facilitate analysis of cell-autonomous ZO-1 trafficking, phosphorylation, membrane redistribution, junctional continuity, and pathway manipulation. They also allow direct coupling of molecular measurements to transendothelial electrical resistance or solute permeability. For example, endothelial studies have demonstrated junctional ZO-1 disorganization together with reduced electrical resistance and increased permeability, allowing closer temporal association between molecular and functional changes. Nevertheless, monocultures exclude the circulating, glial, pericytic, and other neurovascular interactions that substantially modify BBB injury *in vivo*, and findings from these systems should therefore not be presented as established mechanisms operating in the intact ischemic brain.

Coculture and multicellular BBB systems partially bridge this gap by incorporating endothelial interactions with astrocytes, pericytes, or both. These models are particularly useful for testing intercellular signaling and for combining endothelial ZO-1 measurements with electrical-resistance or tracer-permeability assays. Multicellular BBB spheroids, for example, permit assessment of ZO-1-associated changes together with penetration of defined molecular-weight tracers. However, these remain *in vitro* systems, and evidence for pathway regulation or junctional rescue within them does not by itself establish *in vivo* efficacy, systemic target engagement, or relevance to regional ischemia/reperfusion.

Functional assays also require careful interpretation. Transendothelial electrical resistance and tracer permeability provide independent evidence of barrier function but are not specific to ZO-1. Changes in these endpoints may reflect claudin-5 or occludin organization, transcellular transport, endothelial survival, or other barrier mechanisms in addition to ZO-1-containing junctions. Likewise, Evans blue, immunoglobulin, dextran, albumin, or small-solute leakage *in vivo* demonstrates BBB dysfunction but does not automatically identify the paracellular route or establish ZO-1 as its mediator. Edema and hemorrhagic transformation are still broader outcomes because they additionally reflect water transport, extracellular-matrix integrity, proteolysis, vascular injury, and hemostatic processes.

Ultrastructural assessment provides valuable confirmation of tight-junction morphology, endothelial injury, basal-lamina preservation, or perivascular changes, but it does not identify ZO-1 molecular abundance or demonstrate that ZO-1 caused the structural phenotype. Conversely, transcript and protein assays can identify molecular regulation but cannot substitute for ultrastructural or permeability measurements. The most informative studies therefore combine complementary levels of analysis: endothelial or vascular-specific ZO-1 localization/continuity, quantitative molecular measurements, an independent functional barrier endpoint, and experimental manipulation that tests pathway direction or ZO-1 dependence.

Accordingly, this review assigns different interpretive weight to apparently similar statements of “ZO-1 loss” or “ZO-1 restoration.” Tissue-level changes in TJP1 or total ZO-1 are interpreted primarily as abundance-level evidence; vascular immunofluorescence demonstrating redistribution or discontinuity provides spatial evidence; TEER or tracer permeability provides functional evidence; and direct genetic or pharmacological manipulation determines the strength of causal inference. Concordance across these levels provides substantially stronger evidence for ZO-1-associated junctional dysfunction or rescue than any single measurement alone.

## 6 Experimental modulation of ZO-1-associated barrier integrity: Mechanistic rescue or downstream normalization?

The interventions reviewed in this section should be regarded primarily as experimental strategies that modulate ZO-1-associated barrier phenotypes rather than as validated ZO-1-directed therapies. Most act through upstream mechanisms, including inflammatory and proteolytic signaling, oxidative and metabolic stress, cytoskeletal and membrane regulation, growth-factor pathways, or broader neurovascular-unit protection, with preservation or recovery of ZO-1 occurring as one component of the response. Their interpretation therefore follows the evidence framework outlined in the Introduction: greatest mechanistic weight is assigned to direct ZO-1 manipulation, genetic or pharmacological perturbation of an upstream pathway, bidirectional effects, and rescue or reversal experiments, whereas concurrent improvement of ZO-1 and BBB function without experimental linkage is considered pathway-associated or parallel recovery rather than evidence of direct ZO-1 targeting. Interpretation also follows the state-based framework in Table 1 and the model/method framework in Table 2. Recovery of TJP1 transcription or total ZO-1 abundance is distinguished from restoration of endothelial localization or junctional continuity, and tissue-level, spatial, and functional readouts are assigned different interpretive weight. Accordingly, evidence for ZO-1-associated barrier rescue is considered strongest when endothelial or junctional ZO-1 measurements are coupled to an independent functional BBB endpoint and experimental manipulation of the relevant pathway or ZO-1 itself.

Intervention timing is considered explicitly throughout this section. Studies using pretreatment or preconditioning are interpreted as evidence of preventive junctional preservation, whereas interventions initiated during ischemia, at reperfusion, or after reperfusion are distinguished as peri-ischemic protection or post-ischemic rescue, respectively. Accordingly, the term “restoration” is reserved for recovery demonstrated after ischemic or reperfusion-associated junctional disruption rather than used as a general descriptor for all experimental interventions.

### 6.1 Proximal stabilization of junctional trafficking, cytoskeletal, and membrane machinery

Evidence for proximal stabilization of ZO-1-containing junctions was most informative when interventions resolved junctional organization or experimentally manipulated candidate upstream targets (Table 3). Sodium–hydrogen exchanger-1 inhibition with sabiporide preserved continuous membrane-associated ZO-1 and occludin during aglycemic hypoxia despite unchanged total ZO-1, while reducing paracellular and macromolecular permeability. This finding supports preservation of pre-existing junctional organization rather than increased ZO-1 expression, although calcium dependence and ZO-1 trafficking were not directly demonstrated (Park et al., 2010).

**TABLE 3:** Therapeutic proximal stabilization of ZO-1-associated junctional trafficking, cytoskeletal, and membrane machinery after cerebral ischemia.

Intervention	Experimental model	ZO-1 outcome	BBB outcome	Mechanistic interpretation
Sabiporide (Park et al., 2010)	Transient focal cerebral ischemia–reperfusion in rats + aglycemic hypoxia in bEnd.3 endothelial cells; endothelial–astrocyte-like coculture used for permeability	Preserved continuous membrane/junctional ZO-1 despite unchanged total ZO-1 protein during hypoxia	↓ sucrose and albumin permeability *in vitro*; ↓ Evans blue leakage *in vivo*	Strongest spatial evidence for junctional stabilization. Sodium–hydrogen exchanger-1 inhibition reduced intracellular Ca^2+^ accumulation and preserved pre-existing ZO-1 organization rather than increasing expression. However, Ca^2+^ was not independently manipulated and ZO-1 redistribution/internalization was not directly tracked
Anagliptin (Li et al., 2021)	6-h OGD/R followed by 24-h reoxygenation in bEnd.3 brain endothelial cells; treatment during reoxygenation	Dose-dependently restored TJP1 mRNA and total ZO-1 protein; MLCK inhibitor ML-7 produced similar restoration	↓ dextran permeability; ML-7 markedly reduced permeability while suppressing MLC-2 phosphorylation	Supports MLCK/MLC-2 contractile-pathway involvement in ZO-1-associated barrier failure. However, ML-7 phenocopied anagliptin rather than proving that anagliptin requires MLCK signaling; ZO-1 localization and necessity were not tested
Muscone (Yang et al., 2026)	1-h transient MCAO followed by 24-h reperfusion in male mice; treatment initiated at reperfusion	Dose-dependently restored total ZO-1 protein and fluorescence with occludin and claudin-5; PKA inhibition with H-89 attenuated restoration	↓ Evans blue leakage; H-89 attenuated barrier protection	Pathway-mediated contractile rescue. Muscone increased PKA/RHOA phosphorylation, reduced MLC phosphorylation and endothelial apoptosis, while H-89 reversed molecular and functional protection. Lack of an ischemia + H-89-only group limits pathway specificity, and direct ZO-1 redistribution was not examined
Sevoflurane (Zhang et al., 2025)	2-h transient MCAO/reperfusion in rats + OGD/R in rat brain slices; combined pre-, peri-, and postconditioning	Restored ZO-1 protein; TMEM16A overexpression reduced ZO-1 and weakened rescue, whereas TMEM16A knockdown increased ZO-1	↓ fluorescent-tracer leakage and brain edema; preserved vascular ultrastructure; TMEM16A manipulation altered these outcomes bidirectionally	bidirectional TMEM16A manipulation altered ZO-1-associated barrier outcomes; endothelial specificity and ZO-1 localization were not established
Magnolol (Liu et al., 2017)	90-min transient MCAO/reperfusion in mice + primary endothelial and endothelial–pericyte–astrocyte BBB models	Restored total endothelial ZO-1 and occludin *in vitro* while reducing EphA2 phosphorylation; ZO-1 was not measured *in vivo*	↑ TEER; ↓ horseradish-peroxidase permeability *in vitro*; ↓ Evans blue leakage and edema *in vivo*	Supports EphA2-phosphorylation-associated junctional rescue, but the proposed EphA2→ZO-1 sequence was not experimentally interrupted. Because ZO-1 was not measured *in vivo*, animal BBB protection cannot be directly attributed to ZO-1 preservation
Tongxinluo (Chang et al., 2020) with receptor-associated protein as an LRP1 pathway probe	Permanent MCAO in male mice; Tongxinluo initiated after ischemia	Restored total peri-infarct ZO-1, occludin, and claudin-5; receptor-associated protein produced similar recovery	↓ Evans blue leakage at 6 and 24 h	Pharmacological LRP1-associated rescue. LRP1 inhibition phenocopied Tongxinluo for junctional proteins and leakage, supporting pathway involvement. However, no rescue/overexpression experiment demonstrated LRP1 necessity, and ZO-1 was measured at tissue level without localization or continuity assessment
Retigabine (Zhao et al., 2018)	2-h transient MCAO/reperfusion in male rats; treatment 1 h after ischemia onset and before reperfusion; outcomes at 3–96 h	Increased vessel-associated ZO-1 staining and vascular distribution/continuity with occludin and claudin-5 across reperfusion phases	↓ Evans blue extravasation; restored tight-junction ultrastructure	Demonstrates longitudinal junctional preservation, accompanied by lower PKC-δ and MMP-2/9. However, these pathways were not selectively manipulated, MMP activity was not measured, and dependence on retigabine’s Kv7/KCNQ potassium-channel action was not demonstrated

Evidence categories follow the framework defined in the Introduction and Table 1. Unless direct TJP1/ZO-1 manipulation was performed, pathway-supported or associative findings should not be interpreted as demonstrating ZO-1 necessity for BBB protection. Administration timing is reported explicitly where available. Pretreatment or preconditioning is classified as preventive protection; treatment during ischemia or at reperfusion as peri-ischemic/early protection; and treatment initiated after ischemic or reperfusion injury as post-ischemic rescue. These categories describe experimental timing and do not imply equivalent clinical translatability.

Contractile-pathway studies similarly implicated MLCK/MLC and PKA-associated signaling: pharmacological MLCK inhibition reproduced barrier protection in endothelial cells, whereas PKA inhibition attenuated muscone-associated recovery of ZO-1 and BBB integrity *in vivo* (Li et al., 2021; Yang et al., 2026).

Bidirectional manipulation of TMEM16A provided stronger target-level evidence, because its overexpression reduced ZO-1 and weakened sevoflurane-associated BBB protection, whereas knockdown produced the opposite molecular and functional effects. However, endothelial specificity, ZO-1 localization, and the proposed upstream calcium/calmodulin-dependent protein kinase II relationship remain unresolved (Zhang et al., 2025). Other interventions, including magnolol, Tongxinluo, and retigabine, restored ZO-1-containing junctional markers together with improved barrier outcomes, but pathway dependence was not sufficiently established to distinguish direct junctional regulation from broader vascular protection (Liu et al., 2017; Chang et al., 2020; Zhao et al., 2018) (Table 3). Overall, these studies support modulation of membrane, contractile, and ion-channel-associated machinery as upstream determinants of ZO-1-containing junctional stability, but they do not establish ZO-1 as the necessary mediator of barrier rescue.

### 6.2 Anti-inflammatory and anti-proteolytic rescue of ZO-1-containing junctions

Anti-inflammatory and anti-proteolytic interventions frequently preserved ZO-1 alongside reduced BBB leakage, but mechanistic confidence was greatest when pathway perturbation or protease activity measurements accompanied functional barrier assessment (Table 4). Dexmedetomidine reduced MMP-9 activity and inflammatory signaling, while p38 inhibition reproduced barrier protection and JNK or p38 reactivation reversed functional benefits; however, ZO-1 was not reassessed after pathway reactivation, limiting attribution of its restoration specifically to these kinases (Zhu et al., 2024). Doxycycline provided complementary longitudinal evidence by reducing MMP-2/9 activity while preserving microvascular ZO-1 abundance and continuity across early and delayed permeability phases (Wang et al., 2012). Alogliptin and adjudin likewise combined ZO-1 recovery with measurement of MMP activity, although direct proteolysis of ZO-1 was not demonstrated (Hao et al., 2019; Liu T. et al., 2014).

**TABLE 4:** Anti-inflammatory and anti-proteolytic rescue of ZO-1-containing junctions after cerebral ischemia.

Intervention	Experimental model	ZO-1 outcome	BBB outcome	Mechanistic interpretation
Fasudil dichloroacetate (FDCA) (Guan et al., 2023)	Transient/permanent MCAO in rats + OGD in bEnd.3 endothelial cells	Restored total ZO-1 and occludin in peri-infarct tissue and endothelial cells	↓ Evans blue leakage and cerebral edema	Reduced ROCK-associated MYPT1 phosphorylation and MMP-9 alongside ZO-1 recovery. Supports pathway-mediated rescue, but the ROCK/MYPT1→ZO-1 sequence was not independently tested and metabolic/microglial effects were not directly connected to endothelial ZO-1
Dexmedetomidine (Zhu et al., 2024)	1.5-h transient MCAO/R in rats; treatment initiated during ischemia	Restored total ZO-1, claudin-5, and occludin	↓ Evans blue leakage and edema	Strong pathway evidence for BBB protection. P38 inhibition reproduced protection, whereas JNK or p38 reactivation reversed permeability/edema benefits; MMP-9 activity was also reduced. However, ZO-1 was not reassessed after pathway reactivation, so kinase causality for ZO-1 restoration itself remains incomplete
Doxycycline (Wang et al., 2012)	2-h transient MCAO/R in rats; longitudinal assessment from 3 to 120 h	Preserved TJP1 mRNA, microvascular ZO-1 protein, and vascular continuity	↓ Evans blue leakage across early and delayed permeability phases	Particularly strong longitudinal anti-proteolytic evidence: reduced MMP-2/9 expression and gelatinolytic activity and PKC-δ while preserving ZO-1. Direct MMP-mediated ZO-1 cleavage and ZO-1 necessity were not demonstrated
Alogliptin (Hao et al., 2019)	MCAO/R in mice + OGD/R in primary human brain endothelial cells; predominantly preventive treatment	Preserved TJP1 mRNA, ZO-1 protein, and vascular immunoreactivity	↓ Evans blue leakage and endothelial dextran permeability	MMP/TIMP balance and activity changed with ZO-1 and permeability; MMP dependence was not directly tested.
Icariside II (Liu et al., 2020a)	2-h transient MCAO followed by 3 days reperfusion in rats	Restored total ZO-1, occludin, and claudin-5 in ischemic penumbra	↓ Evans blue leakage	Shifted active MMP-2/9–TIMP-1 balance toward inhibition. Molecular docking suggested MMP binding but did not establish biochemical inhibition; direct ZO-1 proteolysis was not demonstrated
Adjudin (Liu et al., 2014a)	1.5-h transient MCAO/R in mice; post-reperfusion treatment	Partially restored total ZO-1 and ZO-1/CD31 vascular alignment	↓ endogenous IgG leakage	Reduced microglial activation, inflammatory mediators, and MMP-9 activity while preserving endothelial-associated ZO-1. Microglia→endothelium signaling and direct MMP-9→ZO-1 degradation were not established
Carbon monoxide-releasing molecule-3 (CORM-3) (Wang et al., 2018)	60-min transient MCAO/R in mice; administered at reperfusion	Restored endothelial-associated ZO-1 and total ipsilateral ZO-1	↓ Evans blue leakage and brain water content	Reduced microglial activation, inflammatory cytokines, and MMP-9 while also preserving laminin and a pericyte marker. Represents broad neurovascular normalization rather than a demonstrated MMP-9→ZO-1 mechanism
Anti-chemokine-like factor 1 antibody (Kong et al., 2016)	60-min transient MCAO/R in rats; intracerebroventricular treatment immediately after reperfusion	Dose-dependently restored total ZO-1 and occludin	↓ BBB leakage and edema; improved astrocytic end-foot/basal-lamina ultrastructure	Direct neutralization of an upstream inflammatory mediator strengthens pathway evidence, but the proposed MMP-9 intermediate was not independently manipulated and ZO-1 necessity was not tested
XingNaoJing injection (Qu et al., 2019)	2-h transient MCAO/R in rats; pretreatment plus reperfusion dose	Partially restored total ZO-1 and occludin	↓ Evans blue leakage	Reduced NLRP3, caspase-1, and interleukin-1β expression, but inflammasome assembly/activity was not demonstrated and no pathway manipulation was performed. Best interpreted as broad anti-inflammatory preservation, not proven NLRP3-dependent ZO-1 rescue
Recombinant human erythropoietin (Liu et al., 2013)	2-h transient MCAO/R in rats; pretreatment; early and delayed permeability phases assessed	Preserved microvascular TJP1 mRNA, ZO-1 protein, and vascular immunoreactivity	↓ early and delayed Evans blue permeability peaks; improved tight-junction ultrastructure	Strong microvascular and temporal evidence; reduced TNF-α/NF-κB accompanied ZO-1 recovery. However, erythropoietin-receptor and inflammatory-pathway causality were not tested, and treatment was prophylactic
Ulinastatin (Li et al., 2018b)	60-min transient MCAO/R in mice; administered immediately after reperfusion	Dose-dependently preserved TJP1 mRNA and total ZO-1 protein	↓ Evans blue leakage and cerebral edema	Post-reperfusion treatment reduced MMP-9 mRNA/protein while restoring ZO-1 and occludin. Supports MMP-9-associated rescue, but MMP activity, direct cleavage, and pathway causality were not tested
Luteolin-7-O-β-D-glucuronide (Fan et al., 2024b)	2-h transient MCAO/R in rats; single dose immediately after reperfusion	Restored total cortical ZO-1; response was not strictly dose-dependent	↓ Evans blue leakage and edema at effective doses; preserved tight-junction/basement-membrane ultrastructure	Reduced MMP-9 and inflammatory cytokines, but no pathway component was selectively manipulated. Importantly, the lowest dose restored ZO-1 without significantly improving leakage or edema, showing that total ZO-1 recovery alone did not ensure functional BBB rescue
Parecoxib (Liu et al., 2020b)	Transient global cerebral ischemia–reperfusion in rats; repeated post-reperfusion treatment	Dose-dependently restored total cortical ZO-1	↓ Evans blue leakage and cerebral edema	Suppressed COX-2/PGE2, TNF-α, IL-1β, and MMP-9 alongside ZO-1 recovery. However, the proposed COX-2→inflammation→MMP-9→ZO-1 sequence was not experimentally interrupted
Tanshinone IIA (Tang et al., 2010)	2-h transient MCAO/R in rats; treatment shortly after ischemia onset	Dose-dependently preserved total ZO-1 and occludin	↓ Evans blue leakage; higher doses ↓ edema	Suppressed ICAM-1 and MMP-9 alongside junctional rescue. MMP-9 activity and direct ZO-1 proteolysis were not assessed; thus, evidence supports broad inflammatory/proteolytic pathway-associated rescue
IMM-H004 (Niu et al., 2017)	1-h transient MCAO/R in rats; treatment timing reported inconsistently	Dose-dependently restored cortical and hippocampal ZO-1 and occludin	↓ Evans blue leakage and edema; preserved endothelial/perivascular ultrastructure	ZO-1 rescue accompanied reduced MMP-9 and AQP4, but MMP-9 activity was not measured and the mechanism was not selectively tested. Broad reductions in infarction also allow secondary junctional preservation
Zafirlukast (Zeng et al., 2020)	MCAO/R in mice + OGD/R in primary human brain endothelial cells; 14-day animal pretreatment	Preserved TJP1 mRNA, endothelial ZO-1 protein, and brain ZO-1 immunofluorescence	↓ Evans blue leakage and endothelial dextran flux	Suppressed NF-κB signaling and MMP-2/9 expression, but CysLT1 receptor dependence, NF-κB causality, MMP dependence, and direct ZO-1 cleavage were all untested. Strongly prophylactic design further limits therapeutic interpretation
Safinamide (Xu et al., 2020)	MCAO/R in mice + OGD/R in bEND.3 endothelial cells; prolonged pretreatment	preserved vessel-associated and endothelial ZO-1 and occludin	↓ Evans blue and endothelial dextran permeability	Reduced MMP-2/9 expression and improved endothelial survival, but protease activity and the proposed monoamine oxidase-B/ion-channel mechanisms were not tested. Best classified as downstream normalization after broader protection
Curcumin (Wu et al., 2021)	90-min transient MCAO/R in rats; pretreatment	Preserved total peri-ischemic ZO-1, occludin, and claudin-5	↓ Evans blue leakage	Reduced phosphorylated NF-κB p65 and MMP-9, but neither pathway was selectively manipulated and no MMP activity/ZO-1 cleavage assay was performed. Represents preventive broad anti-inflammatory/anti-proteolytic protection
Repulsive guidance molecule A (RGMa) silencing (Li et al., 2018a)	2-h transient MCAO/R in rats; adenoviral shRNA delivered immediately after reperfusion	Restored total cortical ZO-1 and claudin-5	↓ Evans blue leakage; improved tight-junction and basement-membrane ultrastructure	Strong genetic upstream evidence. RGMa silencing restored Cdc42/PAK1 abundance and reduced MMP-9, but pathway activity was not measured and Cdc42/PAK1 dependence was not tested. Thus, RGMa is implicated causally in the BBB phenotype, but the downstream route to ZO-1 remains unresolved
Hydroquinone (Ha et al., 2016)	2-h transient MCAO/R in rats; single post-reperfusion dose	Preserved total ZO-1 and continuous ZO-1 distribution along SMI-71-positive cortical microvessels	↓ early Evans blue leakage	Provides strong temporally matched spatial + functional rescue, but no upstream inflammatory, proteolytic, oxidative, trafficking, or cytoskeletal mechanism was measured. Claims of preventing ZO-1 “degradation” therefore exceed the evidence; the high dose also limits translational relevance

Evidence categories follow the framework defined in the Introduction and Table 1. Unless direct TJP1/ZO-1 manipulation was performed, pathway-supported or associative findings should not be interpreted as demonstrating ZO-1 necessity for BBB protection. Administration timing is reported explicitly where available. Pretreatment or preconditioning is classified as preventive protection; treatment during ischemia or at reperfusion as peri-ischemic/early protection; and treatment initiated after ischemic or reperfusion injury as post-ischemic rescue. These categories describe experimental timing and do not imply equivalent clinical translatability.

Genetic or direct mediator manipulation provided additional pathway-level evidence. Post-reperfusion silencing of repulsive guidance molecule A restored ZO-1 and claudin-5, improved junctional and basal-lamina ultrastructure, and reduced leakage, supporting an upstream contribution to the ZO-1-associated BBB phenotype without establishing endothelial specificity or direct ZO-1 regulation (Li M. et al., 2018). Other anti-inflammatory, anti-proteolytic, and preventive interventions produced broadly concordant recovery of ZO-1 and barrier function but generally relied on parallel changes in inflammatory mediators, MMP expression, or tissue-level ZO-1 and are therefore summarized in Table 4 (Guan et al., 2023; Liu MB. et al., 2020; Niu et al., 2017; Zeng et al., 2020; Xu et al., 2020; Wu et al., 2021; Ha et al., 2016; Wang et al., 2018; Kong et al., 2016; Qu et al., 2019; Liu et al., 2013; Li XF. et al., 2018; Fan X. et al., 2024; Liu S. et al., 2020; Tang et al., 2010). One informative exception was luteolin-7-O-β-D-glucuronide, for which the lowest dose restored total ZO-1 without significantly improving permeability or edema, further demonstrating that recovery of ZO-1 abundance alone is insufficient to establish functional BBB restoration (Fan X. et al., 2024).

Collectively, these studies support inflammatory and proteolytic signaling as important upstream determinants of ZO-1-containing junctional injury. Evidence is stronger for pathway-dependent reduction of the injurious inflammatory or proteolytic environment than for direct MMP-mediated cleavage or specific stabilization of ZO-1. No study in this group established that ZO-1 restoration was necessary for the permeability benefit; consequently, these interventions are better interpreted as promoting conditions permissive for junctional recovery than as directly rebuilding or targeting ZO-1.

### 6.3 Antioxidant, metabolic, and cell-death-directed rescue

The strongest BBB-specific antioxidant evidence came from endothelial OGD experiments. Curcumin increased HO-1, restored ZO-1 and occludin abundance and junctional continuity, increased TEER, and reduced horseradish-peroxidase flux. HO-1 inhibition weakened both junctional and functional protection, although genetic validation and ZO-1 necessity were not tested (Wang et al., 2013).

Leonurine provided stronger pathway ordering through the histone deacetylase 4 (HDAC4)–NADPH oxidase 4 (NOX4)–ROS axis. Treatment initiated shortly after reperfusion restored TJP1 mRNA, ZO-1 protein, and endothelial junctional staining while reducing MMP-9, stress fibres, leakage, and edema. Genetic and pharmacological manipulations positioned HDAC4 upstream of oxidant production and ZO-1 loss, but direct ZO-1 regulation and necessity remained untested (Zhang et al., 2017). Procyanidin B2 restored NRF2-associated antioxidant signaling, reduced mitochondrial dysfunction, ROS, and lipid peroxidation, and preserved both total ZO-1 and its continuity along CD31-positive microvessels. These changes accompanied reduced Evans blue and IgG leakage and edema, although pathway necessity was not tested (Wu et al., 2015).

Nomilin pretreatment increased NRF2 and NAD(P)H quinone dehydrogenase 1, improved antioxidant activity, preserved total ZO-1, and reduced leakage and edema. NRF2 knockdown confirmed pathway dependence only for neuronal antioxidant protection, not for endothelial ZO-1 or BBB rescue (Shi et al., 2019). Z-ligustilide pretreatment preserved ZO-1, occludin, and collagen IV while reducing MMP-2/9, leakage, and edema. Inhibition of NRF2-driven transcription or heat shock protein 70 weakened general neuroprotection and anti-apoptotic effects, but BBB and ZO-1 outcomes were not reassessed, leaving their specific roles in junctional preservation uncertain (Li J. et al., 2017).

Metabolic lipid signaling also influenced ZO-1-associated barrier recovery. Soluble epoxide hydrolase inhibition restored ZO-1, claudin-5, and occludin in ischemic tissue and human brain endothelial cells while reducing ROS, inflammation, apoptosis-associated proteins, MMP-2/9, leakage, edema, and endothelial permeability. However, target engagement was not confirmed, and greater ZO-1 abundance did not consistently produce stronger functional protection (Yi et al., 2020). Inhibition of 20-hydroxy eicosatetraenoic-acid synthesis reduced superoxide, JNK/c-Jun phosphorylation, and MMP-9 while restoring ZO-1 and claudin-5. Treatment also reduced Evans blue and sodium-fluorescein leakage, preserved microvascular ultrastructure, and limited delayed edema, although the proposed redox–kinase–protease sequence was not experimentally ordered (Liu Y. et al., 2014).

A peroxynitrite-triggered carbon monoxide donor administered at reperfusion reduced MMP-9, restored total ZO-1, and decreased Evans blue leakage. A peroxynitrite decomposition catalyst reproduced the molecular effects, supporting nitrosative-stress involvement. However, the reported anti-apoptotic and anti-ferroptotic mechanisms were demonstrated mainly in neurons and were not directly connected to endothelial ZO-1, while the specific contribution of carbon monoxide remained unresolved (Guo et al., 2024). Geniposide restored endothelial ZO-1, occludin, and claudin-5 in an astrocyte–endothelial co-culture model, increased electrical resistance, and reduced sodium-fluorescein permeability. These effects accompanied lower oxidative injury, inflammatory cytokines, MMP-2/9, and endothelial apoptosis, but no individual pathway was selectively tested; therefore, ZO-1 recovery may have reflected broader endothelial survival (Li C. et al., 2019).

Antioxidant, metabolic, and cell-death-directed interventions converge on ZO-1 primarily by reducing the cellular stresses that destabilize endothelial junctions. HO-1 inhibition and directional manipulation of the HDAC4–NOX4 pathway provide the clearest pathway-level evidence, whereas NRF2-, lipid-metabolic-, apoptotic-, and ferroptotic mechanisms are generally less specifically linked to endothelial ZO-1 regulation.

### 6.4 Wnt/PI3K/Akt, growth-factor, and reparative signaling

The most direct evidence showed that PI3K/AKT signaling can restore ZO-1 transcription rather than merely normalize it after general tissue protection. KY-226 administered after reperfusion increased TJP1 promoter activity, mRNA, total ZO-1, and vascular-associated expression while reducing Evans blue leakage. PI3K or extracellular signal-regulated kinase inhibition abolished the recovery of TJP1 mRNA and ZO-1 protein, whereas FOXO1 inhibition and overexpression oppositely regulated the TJP1 promoter. These findings identify AKT-dependent phosphorylation and inhibition of FOXO1 as a mechanism relieving transcriptional repression of ZO-1, although direct promoter binding and functional BBB dependence on restored ZO-1 were not demonstrated (Sun et al., 2019).

Canonical Wnt/β-catenin signaling provided the strongest evidence for functional endothelial repair. Lithium administered at reperfusion restored total and vessel-associated ZO-1 and claudin-5, increased endothelial active β-catenin and Wnt transcriptional activity, improved endothelial resistance, and reduced Evans blue and IgG leakage. Conditional endothelial deletion of the Wnt7 co-receptor G-protein-coupled receptor 124 abolished lithium-mediated BBB protection, establishing endothelial Wnt-pathway necessity. However, ZO-1 was not measured after receptor deletion, so the genetic experiment demonstrated pathway-dependent BBB sealing rather than Wnt-dependent ZO-1 restoration specifically (Ji et al., 2021).

Quercetin pretreatment similarly increased β-catenin-associated signaling, preserved ZO-1 and claudin-5, reduced MMP-9, and improved leakage, edema, and tight-junction ultrastructure after global ischemia. The Wnt inhibitor Dickkopf-1 reversed these molecular and functional effects, supporting pathway involvement. Nevertheless, the absence of a Dickkopf-1-only ischemia group, endothelial-specific signaling measurements, and ZO-1 manipulation limits causal interpretation (Jin et al., 2019b). DL-3-n-butylphthalide provided supportive but associative evidence linking AKT, GSK-3β, and β-catenin to junctional restoration. During chronic cerebral hypoperfusion and endothelial hypoxic injury, treatment reduced ROS, increased AKT and inhibitory GSK-3β phosphorylation, increased β-catenin, and restored microvascular ZO-1 abundance and linear endothelial distribution. These effects accompanied reduced Evans blue leakage and improved junctional ultrastructure, but no pathway blockade established that AKT/β-catenin signaling mediated ZO-1 rescue (Ye et al., 2019).

Growth-factor signaling showed context-dependent effects. Suppression of VEGF/VEGF receptor two signaling by juglanin preserved ZO-1 and occludin and reduced brain and endothelial permeability. Exogenous VEGF-A reversed both ZO-1 restoration and the permeability benefit, providing rescue evidence that excessive VEGF signaling contributed to junctional injury; however, the 3-week pretreatment design did not distinguish acute barrier disruption from longer-term angiogenic effects (Liu J. et al., 2020).

In contrast, epidermal growth factor added at reoxygenation partially restored endothelial TJP1 mRNA and ZO-1 protein and reduced Lucifer-yellow permeability without significantly changing cell viability. This supports a post-ischemic reparative effect independent of simple preservation of endothelial-cell number, but epidermal growth factor receptor, AKT, and mitogen-activated protein kinase signaling were not assessed (Yang S. et al., 2019). Continuous hepatocyte growth factor delivery initiated shortly after cerebral embolism preserved ZO-1- and occludin-positive endothelial vessels and reduced fluorescein isothiocyanate–albumin leakage. The spatial association between retained ZO-1 and non-leaky vessels supports junctional preservation, but total ZO-1 abundance, receptor signaling, and proposed VEGF or angiopoietin intermediates were not examined (Date et al., 2006).

Wnt/PI3K/AKT and growth-factor pathways regulate ZO-1 through mechanistically distinct routes. AKT–FOXO1 signaling provides comparatively strong evidence for transcriptional control of TJP1, endothelial GPR124–Wnt/β-catenin signaling supports pathway-dependent BBB repair, and VEGF reversal experiments demonstrate the context-dependent effects of growth-factor signaling on junctional integrity.

### 6.5 Convergent multitarget modulation of ZO-1-associated barrier integrity

Multitarget formulations generally improved ZO-1-associated BBB outcomes, but their mechanistic interpretability depended on whether upstream pathways or active components were experimentally interrogated (Table 5). Guanxinning injection provided comparatively strong pathway-supported evidence by restoring TJP1/ZO-1 and barrier function in multicellular BBB spheroids and ischemic mice, while MMP-9 inhibition reproduced ZO-1 recovery and salvianolic acid B showed direct MMP-9 binding. Nevertheless, direct ZO-1 cleavage, MMP-9 enzymatic dependence, and ZO-1 necessity were not demonstrated (Fan et al., 2025). Shengui Sansheng Pulvis also provided pathway-level evidence because vasoactive intestinal peptide antagonism impaired ZO-1 and barrier function and the formulation partially rescued these effects, although the responsible downstream junctional mechanism remained unresolved (Xia et al., 2020). Weisheng-Tang implicated PAR-1/MMP-9 signaling through agonist reversal experiments, but ZO-1 and permeability were not reassessed after PAR-1 activation, preventing confirmation of a PAR-1–MMP-9–ZO-1 sequence (Kim et al., 2019). Other formulations restored ZO-1 abundance, endothelial distribution, or junctional morphology together with improved barrier-associated outcomes, but pathway-specific manipulation was absent or incomplete; their individual experimental findings are therefore retained in Table 3 rather than repeated here (Zhang Y. et al., 2020; Zhou et al., 2025; Wei et al., 2025; Zhang et al., 2024; Li L. et al., 2017; Qu et al., 2009). YiQiFuMai provides a particularly relevant example of the limitations of abundance-based interpretation: BBB leakage and brain water content improved at doses that did not significantly restore total ZO-1, whereas clear ZO-1 recovery occurred only at the highest dose. Thus, functional BBB protection was not proportional to ZO-1 abundance (Cao et al., 2016), Overall, the evidence places these interventions on a spectrum from pathway-supported junctional rescue to broad downstream neurovascular normalization. Their multitarget actions may preserve conditions favorable to ZO-1-containing junctions, but most studies do not identify a specific molecular route controlling ZO-1, and none demonstrates that ZO-1 is necessary for the observed BBB protection. Detailed study-level differences in experimental model, ZO-1 endpoint, BBB outcome, and mechanistic support are summarized in Table 5.

**TABLE 5:** Multitarget natural products and traditional formulations affecting ZO-1-associated blood–brain barrier integrity after cerebral ischemia.

Intervention	Experimental model	ZO-1 outcome	BBB outcome	Mechanistic interpretation
Guanxinning injection (Fan et al., 2025)	Transient MCAO/R in mice + OGD/R in human endothelial–astrocyte–pericyte BBB spheroids	Preserved TJP1 mRNA and ZO-1 fluorescence; MMP-9 inhibition also restored ZO-1	↓ 10- and 70-kDa dextran permeability; ↓ Evans blue leakage; preservation of basement-membrane proteins	MMP-9 inhibition reproduced ZO-1 recovery; direct ZO-1 cleavage was not demonstrated
Shengui Sansheng Pulvis (Xia et al., 2020)	Permanent MCAO in rats + OGD in primary brain microvascular endothelial cells; treatment began before ischemia *in vivo*	Preserved total ZO-1 in brain tissue and endothelial cells	↓ Evans blue leakage and edema; ↑ TEER; ↓ 4.4- and 70-kDa dextran permeability	Pathway-mediated junctional rescue. Vasoactive intestinal peptide (VIP) antagonism impaired ZO-1 and barrier function, and treatment partially rescued these effects, supporting a contributory VIP-dependent mechanism rather than direct ZO-1 targeting
GRS: ginsenoside Rb1 + ruscogenin + schisandrin A (Zhang et al., 2020d)	Transient MCAO/R in mice + OGD/R in bEnd.3 endothelial cells	Restored total ZO-1, endothelial distribution, and junctional continuity	↓ Evans blue leakage and edema; ↓ sodium-fluorescein permeability	ZO-1 recovery accompanied normalization of ROCK1/cofilin, MLC phosphorylation, actin stress fibres, and MMP-2/9, but none of these pathways was selectively manipulated; therefore, regulation remained pathway-associated rather than causal
Danshen–Chuanxiong formula (DS-CX) and active compounds (Zhou et al., 2025)	OGD/R in human endothelial–astrocyte–pericyte 3D BBB spheroids	Preserved TJP1 mRNA and ZO-1 fluorescence; protocatechuic acid, ferulic acid, and senkyunolide I reproduced the effect	↓ 70-kDa dextran penetration	Active-component screening narrowed the formulation’s breadth, but antioxidant, inflammatory, and apoptotic pathways changed in parallel without pathway-specific manipulation; evidence was also limited to an *in vitro* model
Weisheng-Tang (Kim et al., 2019)	Photothrombotic cortical ischemia in mice; preventive treatment before ischemia	Increased CD31-associated ZO-1 and produced a more homogeneous peri-infarct vascular distribution	↓ Evans blue leakage and brain edema	PAR-1/MMP-9 signaling was implicated, but PAR-1 activation was not followed by reassessment of ZO-1 or permeability; thus, the proposed PAR-1→MMP-9→ZO-1 sequence remained incomplete
YiQiFuMai powder injection (Cao et al., 2016)	1-h transient MCAO/R in mice; administered at reperfusion	High dose restored total cortical ZO-1 and improved endothelial distribution/continuity	↓ Evans blue leakage and brain water content at all doses	permeability improved at doses without significant total ZO-1 recovery, indicating dissociation between abundance and barrier function
Shouhui Tongbian Capsules (Wei et al., 2025)	Transient MCAO/R in rats; 5-day pretreatment	Restored total brain ZO-1 and occludin; endothelial localization/continuity not assessed	↓ brain water content; no direct BBB permeability assay	Proposed gut microbiota–SCFA–PPARγ–lipid metabolism/ferroptosis axis was based largely on parallel multi-omics changes. No microbiota depletion, SCFA manipulation, PPARγ blockade, or pathway rescue linked this pathway causally to ZO-1
Ginseng aconitum decoction (Shenfu Tang) (Zhang et al., 2024)	MCAO/R in mice + OGD/R in bEnd.3 endothelial monolayers	Confirmed restoration of endothelial ZO-1 *in vitro*; *in vivo* ZO-1 reporting was contradictory	↓ 70-kDa dextran permeability *in vitro*; ↓ IgG leakage *in vivo*	Fatty-acid oxidation/CPT1A was associated with BBB protection, but CPT1A was not manipulated and was not linked directly to ZO-1. Conclusions regarding ZO-1 are therefore strongest for the endothelial *in vitro* model
Tong-Qiao-Huo-Xue Decoction (Li et al., 2017b)	Filament MCAO in rats; oral treatment for 15 days after ischemia	Increased total ZO-1, occludin, and claudin-5; improved tight-junction ultrastructure	Improved junctional morphology, but no direct permeability assay was reported	MMP-9 and AQP4 decreased in parallel with junctional recovery, but neither pathway was selectively manipulated. Constituents detected in cerebrospinal fluid were not individually tested; therefore, evidence supports structural/molecular rescue rather than confirmed BBB sealing
Astragaloside IV (Qu et al., 2009)	1.5-h transient MCAO/R in rats; treatment initiated during ischemia	Partially restored ZO-1-positive vascular profiles in the ischemic penumbra	↓ Evans blue leakage and lanthanum extravasation; preserved endothelial ultrastructure	Provides functional and structural junctional rescue, but no upstream mechanism was experimentally investigated; it remains unclear whether ZO-1 synthesis, stability, localization, or simply overall endothelial preservation accounted for the effect

Evidence categories follow the framework defined in the Introduction and Table 1. Unless direct TJP1/ZO-1 manipulation was performed, pathway-supported or associative findings should not be interpreted as demonstrating ZO-1 necessity for BBB protection. Administration timing is reported explicitly where available. Pretreatment or preconditioning is classified as preventive protection; treatment during ischemia or at reperfusion as peri-ischemic/early protection; and treatment initiated after ischemic or reperfusion injury as post-ischemic rescue. These categories describe experimental timing and do not imply equivalent clinical translatability.

### 6.6 Multimodal neurovascular protection and vascular remodeling

Physical, preconditioning, and rehabilitation-related interventions influenced ZO-1-associated barrier integrity through broader effects on oxidative stress, astrocyte–vascular interactions, endothelial survival, perfusion, and vascular remodeling rather than through a single defined ZO-1 regulatory pathway. Their effects are therefore considered here as multimodal neurovascular protection and remodeling rather than as direct ZO-1-targeted mechanisms. Continuous theta-burst repetitive transcranial magnetic stimulation initiated 3 h after photothrombotic stroke preserved peri-infarct ZO-1 and its interaction with claudin-5 at days 6 and 22 while reducing dextran, IgG, and Evans blue leakage at day 6. It also improved endothelial survival, perfusion, and neovascularization and altered HIF-1α, platelet-derived growth factor receptor beta, trophic factors, inflammation, and astrocyte polarization. However, none of these pathways was manipulated, so the findings demonstrate durable junctional and vascular repair rather than a defined mechanism controlling ZO-1 (Zong et al., 2020).

Electroacupuncture preconditioning before focal ischemia increased total cortical ZO-1 and claudin-5 while reducing Evans blue leakage and brain water content. These effects accompanied lower superoxide production, reduced NOX4, and fewer AQP4-positive astrocytes. Because neither oxidase four nor AQP4 was selectively manipulated and ZO-1 was measured only in cortical homogenates, the study supports antioxidant and astrocyte-associated protection rather than a demonstrated oxidase 4–ROS–ZO-1 pathway (Jung et al., 2016). Hyperbaric oxygen partially restored total tissue ZO-1 and Cav-1 after global cerebral ischemia–reperfusion and reduced Evans blue leakage and edema at 48 and 72 h. ZO-1 was already increased at 24 h, however, when permeability remained unimproved, showing that total ZO-1 recovery was not sufficient to indicate functional BBB sealing. Cav-1 and ZO-1 were measured only as parallel tissue-level outcomes, and the absence of normobaric oxygen and pressure controls further limited mechanistic interpretation (Li HZ. et al., 2018).

Physical and preconditioning interventions were associated with preservation of ZO-1-containing junctions together with broader antioxidant, vascular, and neurovascular-unit effects. Repetitive transcranial magnetic stimulation provided the most spatially and temporally resolved evidence, whereas electroacupuncture and hyperbaric oxygen primarily linked ZO-1 changes to wider physiological protection.

## 7 Limitations

The principal limitation of the current evidence is that regulation of ZO-1 is considerably better established than its causal contribution to BBB dysfunction. Multiple studies place trafficking, transcriptional, epigenetic, cytoskeletal, inflammatory, oxidative, and proteolytic pathways upstream of changes in ZO-1, and many interventions restore ZO-1 together with reduced permeability. However, these experiments generally manipulate the upstream pathway rather than ZO-1 itself. Consequently, preservation of ZO-1 may represent a causal component of barrier rescue, a permissive condition for junctional recovery, or a downstream consequence of broader endothelial protection. Direct evidence is uncommon: ZO-1 knockdown partially reversed retinoic-acid-mediated endothelial barrier protection, but this dependence was demonstrated *in vitro* rather than in the corresponding *in vivo* hemorrhagic-transformation model. Thus, the evidence supports ZO-1 as a mechanistically regulated component of the ischemic BBB, but does not establish it as the obligatory mediator through which most reported interventions improve barrier function.

A second limitation is the heterogeneity of ZO-1 and BBB readouts across studies. As summarized in Tables 1, 2, TJP1 transcription, total protein abundance, vascular localization, junctional continuity, post-translational or trafficking changes, and functional permeability represent different levels of evidence and cannot be interpreted interchangeably. Tissue-level measurements may obscure endothelial localization, whereas spatial imaging does not by itself establish functional or causal significance. Likewise, permeability, edema, and hemorrhagic transformation reflect overlapping but non-equivalent aspects of BBB dysfunction. This methodological heterogeneity limits direct cross-study comparison and reinforces the need to interpret ZO-1 findings according to both the molecular state measured and the experimental approach used.

Experimental context further restricts comparison and translation. Reperfusion can worsen or restore ZO-1 depending on timing and tissue region, making apparently discordant findings difficult to compare without matched temporal and spatial measurements. Most experimental animals were young and male, while age, sex, hormonal status, and comorbidities were rarely isolated independently. Diabetes, acute hyperglycemia, hyperlipidemia, renal failure, and other systemic conditions were modeled inconsistently, and some studies lacked the comparison groups needed to distinguish a specific comorbidity effect from greater overall ischemic injury. In addition, several therapeutic interventions were administered prophylactically, intracerebroventricularly, or at the onset of reperfusion, providing stronger evidence for preconditioning or immediate vascular protection than for treatment of an established stroke. Together, these limitations constrain direct comparison across studies and the translation of ZO-1-associated mechanisms to clinically established ischemic stroke.

A further limitation is the scarcity of direct human evidence. Although several studies reviewed here used primary human brain endothelial cells or human multicellular BBB models, these systems remain preclinical and cannot establish how endothelial ZO-1 behaves in patients with acute ischemic stroke. Limited clinical studies have measured circulating tight-junction proteins. In a cohort of 458 patients with ischemic stroke, serum occludin, claudin-5, and ZO-1 were evaluated in relation to hemorrhagic transformation, with the claudin-5/ZO-1 ratio among the markers associated with clinical deterioration caused by hemorrhagic transformation (Kazmierski et al., 2012). More recently, a study of 66 patients with ischemic stroke and 24 stroke mimics reported differences in circulating ZO-1, claudin-5, and occludin and found that their combination with the NIHSS improved discrimination between ischemic stroke and stroke mimics (Kakkar et al., 2024). These observations provide preliminary clinical support for tight-junction proteins as circulating biomarkers, but they do not establish endothelial ZO-1 abundance, membrane localization, junctional continuity, or causal involvement in BBB failure. Circulating ZO-1 should therefore not be interpreted as a direct surrogate for the molecular state of ZO-1 at cerebral endothelial junctions. The studies identified for this review also do not provide clinical evidence that specifically manipulating ZO-1 improves BBB integrity or outcome in patients. Consequently, the mechanistic and interventional conclusions of this review should be regarded as predominantly preclinical until validated using patient-derived material, clinically informative BBB measurements, and appropriately designed human studies.

## 8 Future prospects

The most important next step is to determine when ZO-1 is a causal mediator of BBB failure rather than a molecular correlate of endothelial injury. This will require direct, preferably endothelial-specific and temporally controlled manipulation of TJP1 alongside independent measurements of barrier function. Loss-of-function experiments should test whether preventing ZO-1 recovery abolishes the permeability benefit of interventions that otherwise restore the junction, whereas complementary rescue experiments should establish whether restoring ZO-1 can improve barrier function when the upstream ischemic pathway remains active. Applying this approach to mechanisms for which pathway causality is already relatively strong—including caveolin-dependent trafficking, transcriptional regulation, cytoskeletal signaling, and selected reparative pathways—would help distinguish pathways that act through ZO-1 from those in which ZO-1 merely recovers as part of broader endothelial normalization.

Future studies also require a more explicit definition of “ZO-1 rescue.” Whenever feasible, the same experiment should distinguish TJP1 transcription and total protein abundance from endothelial membrane localization, junctional continuity, redistribution, and turnover, while coupling these measurements to functional permeability. Time-resolved analysis is particularly important because restoration of total ZO-1 may precede functional BBB recovery, and reperfusion can have opposing effects according to tissue region and stage of injury. Functional assessment should also distinguish junction-dependent paracellular leakage from transcellular transport and evaluate relevant complementary components such as the basal lamina when edema or macromolecular leakage is used as an endpoint. Such multidimensional measurements would prevent molecular recovery from being interpreted as functional restoration when the two processes remain uncoupled.

Several mechanistic questions identified in this review can now be tested more directly. Evidence for caveolin-associated internalization and endolysosomal regulation should be extended by determining how trafficking changes the junctional pool of ZO-1 over time. Proposed MMP mechanisms require demonstration of whether ZO-1 is directly cleaved or instead decreases secondary to transcriptional suppression, extracellular-matrix injury, or generalized endothelial damage. Similarly, promoter-associated pathways require experiments that establish the necessity of the relevant transcriptional or epigenetic event for endogenous TJP1 regulation. In multicellular models, identifying the macrophage-, microglia-, astrocyte-, or leukocyte-derived signals that ultimately alter endothelial ZO-1 would close the current gap between cell-specific upstream effects and the molecular event at the endothelial junction.

Finally, the feasibility of developing a specifically ZO-1-based therapeutic strategy remains uncertain. The current literature more strongly supports indirect stabilization of ZO-1-containing endothelial junctions through modulation of upstream injury and repair pathways than pharmacological targeting of ZO-1 itself. Before ZO-1 can be considered a therapeutic target in its own right, it will be necessary to establish which aspects of its biology, including expression, trafficking, membrane localization, junctional continuity, or turnover, are causally relevant to barrier rescue and experimentally tractable. 1ncreasing TJP1 transcription or total ZO-1 abundance may not reproduce restoration of continuous junctional organization, while preservation of ZO-1 may be insufficient to normalize BBB function when transcytosis, basal-lamina injury, endothelial loss, or other neurovascular abnormalities persist. A viable ZO-1-directed strategy would therefore require evidence that controlled manipulation of ZO-1 improves barrier function independently of broader upstream protection, together with endothelial specificity and an achievable post-ischemic therapeutic window. Such effects should be validated during clinically relevant reperfusion and recanalization paradigms, in both sexes and across age groups, and in appropriately controlled models of common metabolic and systemic comorbidities. Findings that remain robust under these conditions should subsequently be tested in human multicellular BBB systems capable of resolving endothelial ZO-1 organization and functional permeability. Only after ZO-1 dependence, functional barrier improvement, and translational feasibility are demonstrated across these levels should ZO-1-directed intervention be considered a plausible therapeutic strategy rather than secondary junctional recovery accompanying broader neurovascular protection.

## 9 Conclusion

The accumulated evidence identifies ZO-1 as a dynamically regulated component of the ischemic BBB whose transcription, abundance, localization, trafficking, and junctional continuity can be altered through distinct mechanisms. Spatial disruption of endothelial-border ZO-1 is more informative for paracellular junctional failure than total abundance alone, while overall BBB function additionally depends on transcellular transport, the basal lamina, endothelial viability, and the broader neurovascular unit. Experimental interventions most often preserve ZO-1 indirectly through upstream inflammatory, oxidative, cytoskeletal, metabolic, or reparative pathways rather than by directly targeting ZO-1. Establishing endothelial ZO-1 dependence and validating junctional rescue under clinically relevant reperfusion, comorbidity, and human BBB conditions will be necessary before ZO-1 can be considered a therapeutic target rather than a mechanistically informative component of barrier integrity.
